# Engineered cross-feeding creates inter- and intra-species synthetic yeast communities with enhanced bioproduction

**DOI:** 10.1038/s41467-024-53117-4

**Published:** 2024-10-16

**Authors:** Young-Kyoung Park, Huadong Peng, Piotr Hapeta, Lara Sellés Vidal, Rodrigo Ledesma-Amaro

**Affiliations:** 1 Department of Bioengineering and Centre for Synthetic Biology, Imperial College London, London, UK; 2 Université Paris-Saclay, INRAE, AgroParisTech, Micalis Institute, Jouy-en-Josas, France; 3 Australian Institute of Bioengineering and Nanotechnology, The University of Queensland, Brisbane, Queensland Australia

**Keywords:** Applied microbiology, Metabolic engineering, Synthetic biology

## Abstract

Microorganisms can be engineered to sustainably produce a variety of products including fuels, pharmaceuticals, materials, and food. However, highly engineered strains often result in low production yield, due to undesired effects such as metabolic burden and the toxicity of intermediates. Drawing inspiration from natural ecosystems, the construction of a synthetic community with division of labor can offer advantages for bioproduction. This approach involves dividing specific tasks among community members, thereby enhancing the functionality of each member. In this study, we identify six pairs out of fifteen composed of six auxotrophs of *Yarrowia lipolytica* that spontaneously form robust syntrophic and synergistic communities. We characterize the stability and growth dynamics of these communities. Furthermore, we validate the existence of syntrophic interactions between two yeast species, *Y. lipolytica* and *Saccharomyces cerevisiae*, and find a strain combination, *Δtrp2* and *Δtrp4*, forming a stable syntrophic community between two species. Subsequently, we introduce a 3-hydroxypropionic acid (3-HP) biosynthesis pathway into the syntrophic community by dividing the pathway among different strains. Our results demonstrate improved production of 3-HP in both intra- and interspecies communities compared to monocultures. Our results show the stable formation of synthetic syntrophic communities, and their potential in improving bioproduction processes.

## Abstract (web summary)

Microorganisms can be engineered to sustainably produce a variety of products including fuels, pharmaceuticals, materials, and food. This work reports the design and creation of syntrophic synthetic communities of single and multiple yeast species, which achieve improved bioproduction.

## Introduction

The advances in synthetic biology and metabolic engineering have led to improved biotechnology processes using microorganisms for the production of food, pharmaceuticals, biofuels, and biomaterials. Despite methodological advances in our capacities to improve microbial strains, some commonly found challenges remain, including metabolic burden due to the high level of pathway engineering, cofactor imbalance, or toxicity of intermediates and/or final products. To overcome the drawbacks of engineering single chassis strain, the establishment of synthetic microbial communities by engineering multiple strains that cooperate during the bioprocess has been proposed^1–3^. By dividing the labor among multiple strains, synthetic communities are able to improve the functionality of each member, reduce metabolic burden and engineering complexity, and accomplish high efficiency of production as found in natural consortia^1,2,4^.

In natural communities, there are various cellular interactions that determine the dynamics of the consortia, such as competition, commensalism, mutualism, or neutralism^5^. When it comes to synthetic consortia, designing a proper interaction between members are crucial for constructing a stable and robust synthetic community^2,3^. A type of mutualistic interaction, cross-feeding or syntrophy, requires each population that relies on each other for survival, which can provide stable coexistence by tying together the members in the community^5,6^. One way to achieve cross-feeding is by using co-auxotrophic strains that exchange essential amino acids to allow each other to grow^7,8^.

It has been generally regarded that yeast co-cultures were not as effective as bacterial ones in forming co-auxotrophic communities, except for strains engineered to produce higher amount of amino acids^8^. Recently, we performed high-throughput screening of syntrophic interactions in the model yeast *Saccharomyces cerevisiae* by using yeast knockout library^9,10^. From this study, 49 pairwise auxotroph combinations which is 3.6% of tested pairs were identified to spontaneously form syntrophic communities and some of them were tested for division of labor, leading to improved bioproduction^10^. This finding suggests that cross-feeding-based communities could be formed in other yeast species, including those with high industrial potential.

*Yarrowia lipolytica* has been gaining interests as a host strain for bioproduction of chemicals, fuels, foods, and pharmaceuticals from both academia and industry^11,12^. Advantageous industrial features of this yeast include robustness, stress tolerance, being amenable by synthetic biology tools, and high cell density cultivation. Most research using *Y. lipolytica* have focused on engineering in a single strain. The studies on microbial communities using this yeast are so far limited. There are few studies of co-culture using *Y. lipolytica* with other species for bioremediation or feedstock utilization with the modulation of inoculation ratio or time among community members^13–20^. A study has explored division of labor for bioproduction of amorphadiene with *Y. lipoltyica* strains. A modular co-culture dividing the pathway for boosting precursor pools and amorphadiene synthesis resulted in the improved titers^21^. These works highlight the increasing interest in creating communities of *Y. lipolytica*. However, tools for controlling population dynamics, such as cross-feeding^9^, to maximize robustness and efficiency have not yet been developed in *Y. lipolytica*.

In this study, we explored the creation of syntrophic communities of *Y. lipolytica* using auxotrophic strains and identified pairs exhibiting synergistic growths, which were further characterized. The *Y. lipolytica* auxotrophic strains were also evaluated for establishing the interspecies syntrophic growth with *S. cerevisiae* auxotrophs. We finally developed a division of labor strategy for the production of a bioplastic precursor, 3-hydroxypropionic acid, employing syntrophic intraspecies and interspecies communities, which resulted in increased bioproduction.

## Results

### Establishing synthetic *Y. lipolytica* communities by engineering cross-feeding behaviors

To evaluate if auxotrophs of *Y. lipolytica* could form syntrophic communities by exchanging essential metabolites, we constructed the strains ∆*lys5*, ∆*trp2*, ∆*trp4*, ∆*met5, ∆ura3* and ∆l*eu2*, auxotrophic for lysine, tryptophan, methionine, uracil, and leucine.

**Fig. 1:**
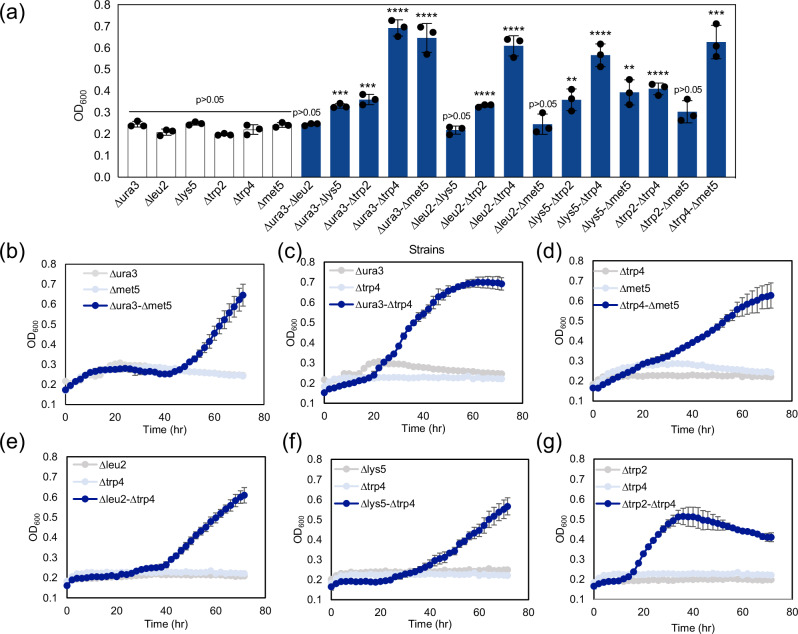
Syntrophic cocultures of *Y. lipolytica* auxotroph strains. **a** OD_600_ values of monocultures (a single auxotroph, empty bars) and cocultures (a pair of two auxotroph with 1:1 inoculation ratio, blue bar) at 72 hours of cultivation. **b**–**g** Growth profile of successful cocultures. Auxotroph monocultures (negative controls, gray and light blue) were tracked along with the corresponding coculture (navy). *N* = 3 biologically independent samples and data are presented as mean ± standard deviation. One-way ANOVA, followed by Bonferroni’s multiple comparisons test with 95% confidence intervals was performed using GraphPad Prism 9.5.0 software and *p* values are indicated as asterisks in the graph (*:*p* < 0.05, **: *p* < 0.005, ***: *p* < 0.0005, ****: *p* < 0.0001). Source data are provided as a Source Data file.

The growth of 15 paired combinations from these six auxotrophs was tested at a 1:1 inoculation ratio in YNBD media without amino acid supplementation. The observed growth of the tested combinations can be grouped into three categories according to their maximal OD_600_ during the cultivation (Fig. 1a, Supplementary Table 1, Supplementary Figs. 1, 2): high (OD_600_ ≧ 0.55): ∆*ura3*-∆*trp4*, ∆*ura3*-∆*met5*, ∆*leu2*-∆*trp4*, ∆*lys5*-∆*trp4*, and ∆*trp4*-∆*met5*; moderate (0.32 ≦ OD_600_ < 0.55): ∆*ura3*-∆*lys5*, ∆*ura3*-∆*trp2*, ∆*leu2*-∆*trp2*, ∆*lys5*-∆*trp2*, ∆*lys5*-∆*met5*, and ∆*trp2*-∆*trp4*; and low (OD_600_ < 0.32): ∆*ura3*-∆*leu2*, ∆*leu2*-∆*lys5*, ∆*leu2*-∆*met5*, and ∆*trp2*-∆*met5*. Among the high-growth combinations, three pairs (∆*leu2*-∆*trp4*, ∆*lys5*-∆*trp4*, and ∆*trp4*-∆*met5*) showed a constant increase in growth, while the other two pairs (∆*ura3*-∆*trp4* and ∆*ura3*-∆*met5*) exhibited an exponential growth after a certain time of lag phase (40 and 20 hours, respectively) (Fig. 1b, Supplementary Fig. 1). Positive correlation between growth and glucose consumption depending on the auxotroph pairs was observed (Supplementary Fig. 3). The shortest lag phase, 12 hours, was found in the combination of ∆*trp2* and ∆*trp4*, and the stationary phase was reached at 36 hours of cultivation. While the final OD_600_ reached by the ∆*trp2* and ∆*trp4* did not rank amongst the top, it exhibited the highest growth rate compared to other combinations (Supplementary Table 1). The prolonged lag phase in some of the synthetic communities could be originated by the needs of each population to adapt their metabolism to export metabolites, which is required by its partner, and/or to import metabolites secreted from the partner^10^.

### Characterization of growth dynamics of synthetic cross-feeding communities

**Fig. 2:**
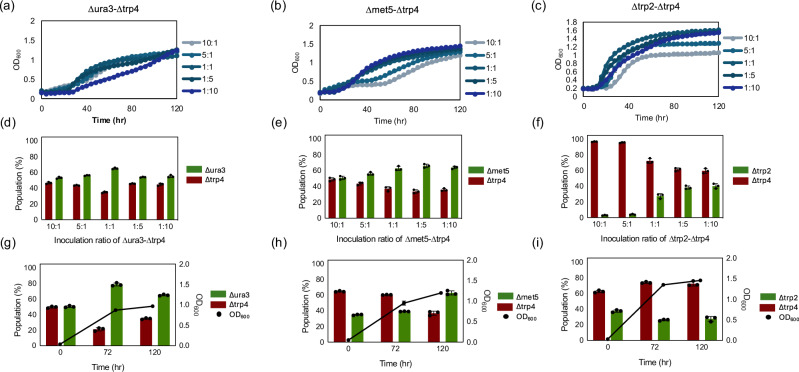
Growth and population of coculture between auxotroph strains with different inoculation ratio. Growth curve from the coculture of (**a**) *Δtrp4-Δura3*, (**b**) *Δtrp4-Δmet5*, and (**c**) *Δtrp2-Δtrp4*. Population ratios reached at stationary phase depending on inoculation ratio in the coculture of (**d**) *Δtrp4-Δura3*, (**e**) *Δtrp4-Δmet5*, and (**f**) *Δtrp2-Δtrp4*. Population dynamics at the 1:1 ratio from the combination of (**g**) *Δtrp4-Δura3*, (**h**) *Δtrp4-Δmet5*, and (**i**) *Δtrp2-Δtrp4*. Populations were measured by flow cytometry. *N* = 3 biologically independent samples and data are presented as mean ± standard deviation. Source data are provided as a Source Data file.

We characterized population dynamics of three selected pairs (∆*ura3*-∆*trp4*, ∆*trp4*-∆*met5*, and ∆*trp2*-∆*trp4*) by varying the inoculation ratios from 10:1 to 1:10 (Fig. 2). Changes in inoculation ratios exhibited considerable differences in growth patterns, which suggest that certain population ratios favor syntrophic growth. In the pair of ∆*ura3*-∆*trp4*, the inoculation ratios of 10:1 and 5:1 showed a shorter lag phase than other ratios, suggesting the importance of having more ∆*ura3* cells at the beginning of the culture (Fig. 2a). Despite the shorter lag phase for these ratios, all inoculation ratios reached a similar final OD_600_ at 120 hours. In addition, regardless of the initial ratio, the population tended to stabilize at the end of the stationary phase, maintaining a ratio of ∆*ura3*:∆*trp4* between 1:1.2 and1:1.8 (Fig. 2d, g, Supplementary Fig. 5). In the case of ∆*met5*-∆*trp4* pair, the coculture with initial ratios of 1:1, 1:5 and 1:10 started growing earlier than those with 10:1 and 5:1 (Fig. 2b). At ratios of 10:1 and 5:1, the cocultures showed a mild growth until 48 hours followed by exponential phase. The final OD_600_ was correlated with the inoculation ratio from 1:10 to 10:1 which also corresponded to glucose consumption (Supplementary Fig. 4). At the stationary phase, the population ratio was stabilized in all cases (∆*met5*:∆*trp4* between 1:1.0 and 1:1.9 (Fig. 2e, h, Supplementary Fig. 6). The pair of ∆trp2-∆*trp4* showed a faster growth at all inoculation ratios compared to other combination tested (Fig. 2c). The ratio 1:10 showed the shortest lag followed by 1:5 and 1:1, while the ratios of 5:1 and 10:1 resulted in a longer lag phase and lower final OD_600_. Since the mutations ∆*trp2* and ∆*trp4* are both mapped in the same tryptophan synthesis pathway, the growth between these two auxotrophs is achieved by the exchange of intermediates, which are known to be anthranilate and indole/tryptophan in *S. cerevisiae*^10^. In *S. cerevisiae*, the coculture of ∆*trp2*-∆*trp4* was naturally highly enriched in one population, ∆*trp2* cells, after inoculating at 1:1 ratio. Similarly, a majority of ∆*trp2* cells were found in the *Y. lipolytica* coculture at 10:1 and 5:1 inoculation ratios, which showed a slower and lower growth compared to other inoculation ratios (Fig. 2f, Supplementary Fig. 7). However, in *Y. lipolytica*, the ratio resulting in better growth exhibited different population dynamics, the ratio of ∆*trp2*:∆*trp4* at the stationary phase was 1:1.5 from the inoculation ratio of 1:5 and 1:10 (Fig. 2i). These considerable distinct population dynamics between two species suggest differences in metabolite exchange rates or mechanisms between *S. cerevisiae* and *Y. lipolytica*.

### Establishing cross-feeding communities between two yeast species, *Y. lipolytica* and *S. cerevisiae*

**Fig. 3:**
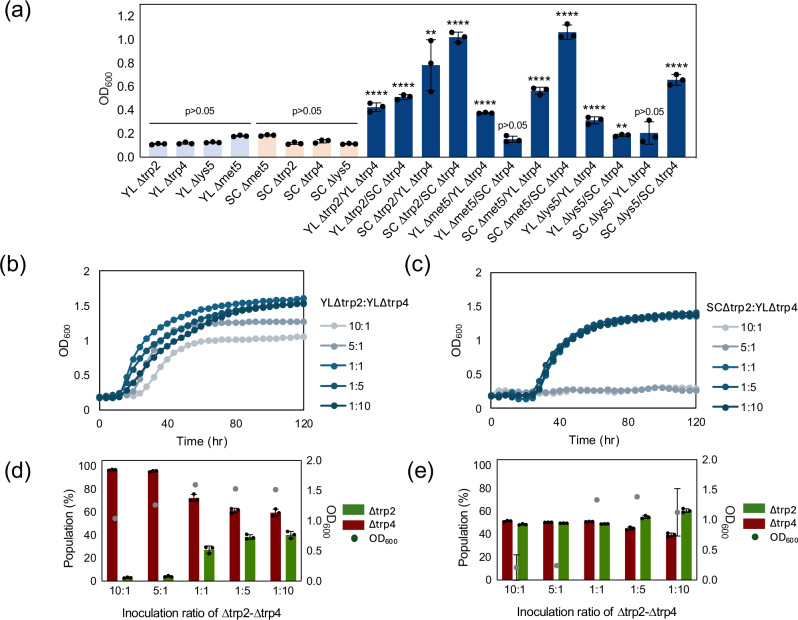
Syntrophic coculture of *Y. lipolytica* (YL) and *S. cerevisiae* (SC) auxotroph strains. **a** OD_600_ values of monocultures (a single auxotroph, light blue for *Y. lipolytica* and light orange for *S. cerevisiae*) and cocultures (pairs of two auxotroph with 1:1 inoculation ratio, blue) at 72 hours of cultivation. **b** Growth of *Y. lipolytica* coculture in the YL∆*trp2*-YL∆*trp4* combination. **c** Growth of *Y. lipolytica* and *S. cerevisiae* coculture in the SC∆*trp2*-YL∆*trp4* combination. **d** population of each strain at different inoculation ratio in YL∆*trp2*-YL∆*trp4* coculture at 120 hours of cultivation. **e** population of each strain at different inoculation ratio in SC∆*trp2*-YL∆*trp4* coculture at 120 hours of cultivation*. N* = 3 biologically independent samples and data are presented as mean ± standard deviation. One-way ANOVA, followed by Bonferroni’s multiple comparisons test with 95% confidence intervals were performed using GraphPad Prism 9.5.0 software and *p* values are indicated as asterisks in the graph (*: *p* < 0.05, **: *p* < 0.005, ***: *p* < 0.0005, ****: *p* < 0.0001). Source data are provided as a Source Data file.

After demonstrating the formation of stable cross-feeding co-cultures between two *Y. lipolytica* auxotrophs, we decided to test whether the syntrophic growth could be established between the two species, *Y. lipolytica* and *S. cerevisiae*. We selected the *Y. lipolytica* auxotrophs described above (YL*Δtrp2*, YL*Δtrp4*, YL*Δmet5*, and YL*Δlys5)* and the *S. cerevisiae* ones based on our previous study (SC*Δtrp2*, SC*Δtrp4*, SC*Δmet5*, and SC*Δlys5)*^10^. Three pairs (YL*Δtrp2*-SC*Δtrp4*, SC*Δtrp2*-YL*Δtrp4*, and SC*Δmet5*-YL*Δtrp4*) showed syntrophic growth in the interspecies coculture (Fig. 3a, Supplementary Fig. 8). The growth dynamics differed depending on the auxotrophies and species involved. For example, the pair of YL*Δmet5*-SC*Δtrp4* did not grow while SC*Δmet5*-YL*Δtrp4* showed higher OD_600_ than the coculture of YL*Δmet5*-YL*Δtrp4*. This might be due to the different metabolite exchange rates among species. As SC*Δtrp2-*YL*Δtrp4* pair showed higher growth compared to other auxotrophic pairs, we further characterized the population dynamics of SC*Δtrp2-*YL*Δtrp4* and YL*Δtrp2-*YL*Δtrp4* by inoculating different ratios (Fig. 3b–e, Supplementary Figs. 9 and 10). Both cocultures showed better growth at 1:1, 1:5, and 1:10 initial ratios. However, the inoculation ratios of 10:1 and 5:1 in SC*Δtrp2-*YL*Δtrp4* failed to grow, which could indicate that the exchange of the intermediate (anthranilate) was not enough for SC*Δtrp2* to grow in these conditions. The populations of SC*Δtrp2-*YL*Δtrp4* stabilized between the ratios of 1:0.9 and 1:1.5 at the stationary phase, which differs from the skewed population distribution of the SC*Δtrp2-*SC*Δtrp4* coculture. To validate whether different cultivation conditions affect the syntrophic growth of SC*Δtrp2*-YL*Δtrp4*, especially regarding a potential influence of the Crabtree effect, co-cultures of SC*Δtrp2*-YL*Δtrp4* with different glucose concentrations (20 and 100 g/L) and aeration condition (aerobic and semi-anaerobic) were performed (Supplementary Fig. 11). At 20 g/L of glucose, the coculture SC*Δtrp2* : YL*Δtrp4* showed growth at 1:1 ratio in semi-anaerobic conditions, while no growth was observed in aerobic conditions. We observed a higher production of ethanol in the 1:1 ratio than in the 1:10 ratio, suggesting the Crabtree effect helped the growth of SC*∆trp2*. At a higher glucose concentration (100 g/L), in both aerobic and semi-anaerobic conditions, we generally observed higher growth when there was a higher presence of the *∆trp4* strain. In the SC-YL co-culture, we observed the Crabtree effect, as reflected by the ethanol production (100 g/L glucose, semi-anaerobic condition) that seemed to come from SC*∆trp2*. As expected, a negligible amount of ethanol was observed in YL-YL co-culture in the same condition.

### Division of labor in cross-feeding communities improves bioproduction

Splitting metabolic pathways between strains in communities (division of labor) can be effective for bioproduction as it can reduce metabolic burden and avoid bottlenecks or toxic intermediates^2^. As a proof of concept, we aimed to synthesize a value-added molecule by splitting the biosynthesis pathway into a cross-feeding community. We selected the Δ*trp2*-Δ*trp4* pair because of its shorter lag phase and more rapid growth (Fig. 1).

**Fig. 4:**
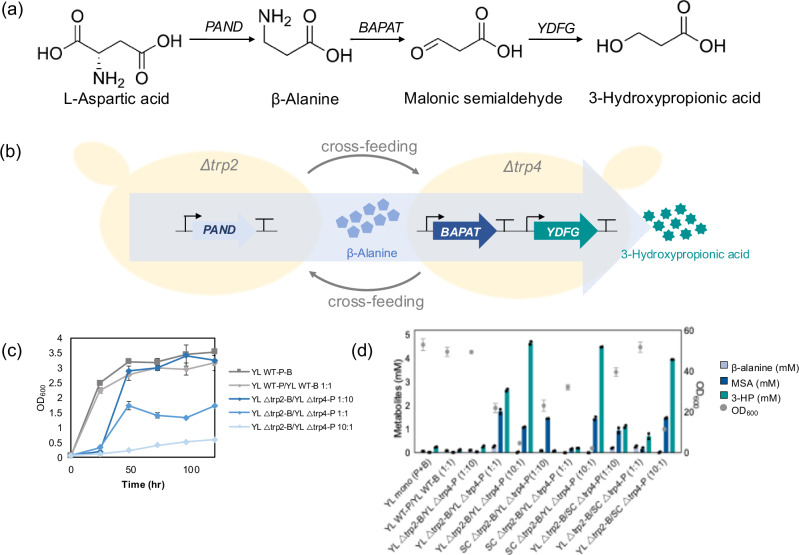
Division of labor in syntrophic community for bioproduction of 3-hydroxypropionic acid. **a** Biosynthetic pathway of 3-hydroxypropionic acid. **b** Strategy of division of labor in the synthetic community. **c** Growth of *Y. lipolytica* intraspecies syntrophic community. **d** Production of metabolites in the 3-HP synthetic pathway from the intraspecies and interspecies synthetic communities. *N* = 2 biologically independent samples and data are presented as mean ± standard deviation. Source data are provided as a Source Data file.

3-Hydroxypropionic acid (3-HP, C_3_H_6_O_3_) is a desired platform chemical with a wide range of applications, as identified by the US Department of Energy in 2004. It is a precursor of acrylic acid, 1, 3-propandiol, malonic acid, biodegradable polyesters, and other valuable chemicals^22,23^. In order to synthesize 3-HP, we selected the biosynthetic pathway through β-alanine and malonic semialdehyde, which has not yet been applied in *Y. lipolytica*. This pathway requires the expression of three enzymes, aspartate-1-decarboxylase (TcPAND from *Tribolium castaneum*), β-alanine-pyruvate aminotransferase (BcBAPAT from *Bacillus cereus*), and 3-hydroxypropanoate dehydrogenase (EcYDFG from *Escherichia coli*) (Fig. 4a)^23^. The 3-HP pathway was split into module P, expressing TcPAND, and module B, expressing BcBAPAT and EcYDFG. Each module is integrated into two distinct auxotrophic strains, thereby generating a community that relies on the transport of β-alanine from one strain to another for producing 3-HP (Fig. 4b).

First, we tested intraspecies *Y. lipolytica* communities with division of labor and compared them with two WT controls, monoculture and coculture (Fig. 4, Supplementary Figs. 12 and 15). The WT monoculture bears three enzymes without division of labor and the WT coculture is composed of two strains, one harboring module P (WT-P) and the other module B (WT-B), thereby implementing division of labor but without cross-feeding. The growth was comparable between the WT monoculture and the WT coculture (Fig. 4c). The production of 3-HP was two times lower in the WT coculture compared to the WT monoculture (Fig. 4d), suggesting that division of labor, without cross-feeding, was not beneficial. In the case of the cross-feeding communities, the growth varied depending on the inoculation ratio of the strains Δ*trp2* with module B (Δ*trp2-*B) and Δ*trp4* with module P (Δ*trp4*-P) (Fig. 4c), which is consistent with the result shown in Fig. 3c. When the initial ratio was 1:10 (Δ*trp2-*B:Δ*trp4*-P), the synthetic community reached a similar OD_600_ as the WT monoculture after 48 hours of cultivation (Fig. 4c). However, the growth of coculture with initial ratios of 1:1 and 10:1 resulted in lower growth. The production of metabolites varied significantly with the inoculation ratio. Coculture at the ratio of 1:10 showed a comparable 3-HP production (0.26 mM) to one from the WT monoculture. The coculture of Δ*trp2-*B and Δ*trp4*-P with an initial ratio of 10:1 reached a production of 4.67 mM of 3-HP, which is 19.3 times higher than the WT monoculture. Instead, WT monoculture produced higher citrate than co-culture (Supplementary Figs. 15 and 16). The higher ratio of module B in the communities showed higher 3-HP production, suggesting that the conversion of β-alanine to 3-HP is more important than the one from L-aspartate to β-alanine for higher production of 3-HP.

As cross-feeding communities were successfully established between *Y. lipolytica* and *S. cerevisiae*, we then decided to study the division of labor within this interspecies community. The strain pairs YL*Δtrp2-*SC*Δtrp4* and SC*Δtrp2-*YL*Δtrp4*, each with different 3-HP synthesis modules, were cultured using different initial inoculation ratios (Fig. 4, Supplementary Fig. 13). Consistently with what was observed for the corresponding cocultures without 3-HP bioproduction modules (Fig. 3c), different growth and metabolite profiles were observed depending on the inoculation ratio (Supplementary Fig. 10). The production of 3-HP varied depending on the combination of species and modules used for 3-HP production. Higher 3-HP production was commonly obtained with the ratio of B:P = 10:1 which is consistent with the result of *Y. lipolytica* intraspecies communities. This also demonstrates an effective transport of β-alanine from the *Δtrp2-*P strain to the *Δtrp4-*B strain in the syntrophic community. The highest 3-HP production from interspecies communities was achieved at 10:1 ratio of SC*Δtrp2*-B*:*YL*Δtrp4*-P, reaching 4.50 mM which is 40.3 and 18.6 times higher than the one from the WT monoculture of *S. cerevisiae* and *Y. lipolytica*, respectively (Fig. 4d, Supplementary Fig. 14). Therefore, these results successfully demonstrated an improvement of 3-HP production through pathway split (and likely division of labor) in both types of synthetic cross-feeding communities, the intraspecies of *Y. lipolytica* and the interspecies of *Y. lipolytica* and *S. cerevisiae*.

## Discussion

In nature, many microorganisms are auxotrophs and therefore rely on external nutrients (including amino acids) for their growth^24^. This observation has inspired synthetic biologists to design synthetic communities using amino acid or nucleotide auxotrophic strains. The requirement on essential metabolites exchange promotes cooperative behaviors and beneficial interactions. Recent studies on synthetic communities often require a high level of engineering to maintain the stability of the coculture and control the population^25,26^, which limit the applicability and universality of these methods. Auxotrophic-based cross-feeding offers a simpler alternative to creating stable communities. However, identifying the adequate pairs of auxotrophs able to establish syntrophic interactions is challenging as metabolic costs and energy requirements for the synthesis of each amino acid or metabolite vary and their transport systems are not fully understood^27–29^.

Here, we aimed to uncover spontaneous syntrophic communities in *Y. lipolytica*. Out of fifteen combinations involving six auxotroph strains, five exhibited robust syntrophic growth, and six demonstrated a slower but still discernible growth at an initial ratio of 1:1. Further investigation by modulating the initial inoculation ratio could potentially unveil additional auxotrophic pairs capable of establishing syntrophic communities. Generally, the success of syntrophic interaction is thought to be determined by the rates of import, export, and consumption of the involved metabolites, as the depletion of one of the metabolites before establishing the syntrophy can lead to the collapse of the community^30^. Therefore, pairs that failed to establish spontaneous syntrophic interactions might be attributed to low production or a limited transport system of specific metabolites that need to be provided to the other member of the community. Engineering strains to overproduce specific metabolites through the regulation of feed-back inhibition or the strengthening carbon flux towards their synthesis could be beneficial in promoting syntrophic interactions, as demonstrated independently in both *E. coli* and *S. cerevisiae*^8,9,31^. At a more fundamental level, it would be beneficial to study the transport mechanisms of metabolites, including amino acids in *Y. lipolytica*. Understanding the secretion or uptake of metabolites is pivotal in order to improve stable syntrophic interactions. Employing omics approaches, such as metagenomic sequencing^7^ and exometabolomic analysis^32,33^ could contribute to unravel some of these transport systems and better understand microbial cross-feeding within synthetic communities.

In this work, we also demonstrated spontaneous syntrophic growth between two yeast species, *Y. lipolytica* and *S. cerevisiae*. A pair of *Δtrp2*-*Δtrp4* demonstrated successful syntrophic interaction between two strains regardless of the combination of the auxotroph pairs and the species. In *S. cerevisiae* communities, *Δtrp2*-*Δtrp4* has been described to exhibit an extremely unbalanced population distribution, with one strain dominating the coculture (over 95% of ∆*trp2*)^10^. A similar trend was observed in the *Y. lipolytica* communities at inoculation ratios of 10:1 and 5:1, however, the population was more balanced (∆*trp2*:∆*trp4* of 1:0.9-1.5) at the ratio of 1:5 and 1:10 (Fig. 2f, i). In the interspecies coculture of *Δtrp2*-*Δtrp4*, a balanced population was achieved in all tested strains, species, and ratios (Fig. 3e), highlighting the potential of interspecies syntrophic communities to provide an additional level of control. In specific inoculation ratios (10:1 and 1:1) of the SC*Δtrp2-*YL*Δtrp4* coculture, growth failed to occur, suggesting an insufficient exchange of metabolites in this experimental condition. Similarly, the pair of YL*Δmet5*-SC*Δtrp4* was unable to grow, while SC*Δmet5*-YL*Δtrp4* grew. This observation might also be explained by different exchange rates of metabolites in different species^7,10^. Interdependent cocultures for bioproduction have so far mostly been explored using model microorganisms^1,34^. Our results suggest a broader applicability of syntrophic interactions beyond model microorganisms, paving the way for designing synthetic communities of non-conventional yeasts for bioproduction.

To study the effect of division of labor and cross-feeding in bioproduction by synthetic communities, we divided the biosynthetic pathway of 3-HP into two modules. The coculture of YLΔ*trp2-*B and YLΔ*trp4*-P, with an initial ratio of 10:1, produced 19.3 times higher 3-HP (4.67 mM) than the WT monoculture harboring the complete 3-HP synthetic pathway in a single strain. Notably, this synthetic pathway converting β-alanine into 3-HP was investigated in *Y. lipolytica* for the first time in this study. The growth and metabolite analysis (Supplementary Fig. 16) suggests that the higher 3-HP production found in the co-cultures originated from a higher availability of pyruvate, a common precursor of 3-HP and citrate. This result underscores that the division of labor within a synthetic community can be used to validate undiscovered synthetic pathways, in addition to the traditional approach of embedding the entire pathway in a single strain. The production of 3-HP was further improved in the interspecies cross-feeding community, YL Δ*trp2-*B and SC Δ*trp4*-P at a 10:1 inoculation ratio, reaching 3.96 mM of 3-HP. This is slightly higher than the reported 0.35 g/L (3.88 mM) of 3-HP production in *Y. lipolytica* harboring the alternative pathway from malonyl-CoA^22^.

When it comes to MSA production in *S. cerevisiae* communities, the coculture of *Δtrp2*-B:*Δtrp4*-P = 10:1 produced the highest MSA among different inoculation ratios but also outperformed the monoculture, which is consistent with the MSA production in a previous study of *S. cerevisiae* communities (Supplementary Table 4 and Supplementary Figs. 14 and 18)^10^. However, the production of MSA in *S. cerevisiae* co-culture at *Δtrp2*-B:*Δtrp4*-P = 1:1 and 1:10 was negligible, although it was higher than the monoculture in the previous study. This might be due to the different promoters used for expressing BAPAT in each study, additional gene expression (YDFG) in this study, and different cultivation scales.

In the case of 3-HP production, *S. cerevisiae* co-culture at specific inoculation ratio (*Δtrp2*-B:*Δtrp4*-P = 1:1) performed better than the *S. cerevisiae* monoculture (Supplementary Fig. 14, Supplementary Table 4). The level of total metabolites produced from the *Δtrp2*-B strain (MSA and 3-HP) in the coculture of *Δtrp2*-B:*Δtrp4*-P = 10:1 is higher than the one from the monoculture (Supplementary Table 4 and Supplementary Fig. 14). In this study, we used the biosynthetic pathway of 3-HP as a proof of concept, but further modifications can lead to improve titers. Increased production is expected through additional engineering strategies such as promoter engineering, multi-copy integration, and precursor and/or cofactor supply. Overall, this work demonstrates that the combination of cross-feeding and inoculation ratio to control population dynamics in synthetic yeast communities with division of labor has the potential to improve the production of valuable molecules. It is worth noting that further research is required to understand the complex relationship between division of labor and bioproduction and fully correlate them both. The strategy described here could be expanded to multiple organisms (and their combination) and compounds of interest.

In conclusion, we successfully demonstrated the establishment of a stable synthetic cross-feeding yeast community employing auxotrophs of *Y. lipolytica*, an yeast of high industrial interest. Synthetic communities of *Y. lipolytica* were characterized in terms of growth and population dynamics, considering different auxotrophic pairs and inoculation ratios. Our findings confirmed that specific auxotrophs can exchange metabolites with other members, facilitating spontaneous growth in both intraspecies (*Y. lipolytica*) and interspecies (*Y. lipolytica* and *S. cerevisiae*) communities. We further explored the division of labor and bioproduction of 3-HP within these syntrophic communities. Notably, we found a 3-HP production improvement by 19.3 and 18.6 times when labor was divided in intra- and interspecies communities compared to the *Y. lipolytica* monoculture, respectively. This study represents the first demonstration of a division of labor for biosynthetic heterologous pathway using syntrophic communities of *Y. lipolytica*. Our findings shed light on the potential of utilizing non-conventional microorganisms to form enhanced synthetic communities for bioproduction of various value-added molecules.

## Methods

### Strains and media

The *E. coli* strains DH5α and TOP10 were used for plasmid construction. *E. coli* strains were grown at 37 °C in Luria−Bertani (LB) medium (containing 1% tryptone, 0.5% yeast extract, and 1% sodium chloride) or on LB agar with appropriate antibiotics.

PCR amplifications were performed in a PCR ProFlex^TM^ (Applied Biosystems) with Q5 High-Fidelity DNA Polymerase (New England Biolabs). PCR fragments were purified with a QIAgen Purification Kit (Qiagen). The plasmids used in this study were constructed by Golden Gate Assembly, as described in Yuzbashev et al.^35^. In brief, each component for GG assembly was cloned to Lv0 plasmid by using BsmBI. Lv1 plasmid containing the specific overhang for Lv2 plasmid was then constructed by assembling the Lv0 plasmid consisting of promoter, gene, and terminator using BsaI. Finally, the Lv2 plasmid containing two or three transcription units was constructed by using BsmBI. To verify the correct construction of plasmids, PCR with GoTaq DNA polymerases (Promega) and digestion by restriction enzyme (New England Biolabs) were carried out.

*Y. lipolytica* was routinely grown at 30 °C in YPD medium which consists of 1% yeast extract, 2% peptone, and 2% glucose, or yeast synthetic medium (YNBD) which includes 0.17% yeast nitrogen base without amino acids and ammonium sulfate, 0.5% ammonium chloride, 50 mM phosphate buffer (KH_2_PO_4_-Na_2_HPO_4_, pH6.8), and 2% glucose. To prepare the solid medium, 1.5% agar was added to the respective liquid medium. To complement auxotrophic processes, uracil, leucine, lysine, methionine, or tryptophan were added at a concentration of 0.1 g/L, as necessary.

To introduce gene expression cassettes into *Y. lipolytica*, NotI-linearized plasmids were transformed into competent cells by the lithium acetate/DTT method. The gene expression cassettes were randomly integrated into the genome of *Y. lipolytica*. Transformants were selected on YNBD media containing the appropriate amino acids for their specific genotype. Positive transformants were then confirmed by colony PCR with Phire Plant Direct PCR master mix (Thermo Fisher). Auxotroph strains were constructed by homologous recombination of promoter and terminator region of marker gene. The resulting auxotroph strains were verified on YNBD media with/without the corresponding amino acids. The removal of the selection marker was carried out via the Cre-LoxP system. The strains and plasmids used in this study are listed in Table 1. The primers used for cloning and verification are listed in Supplementary Table 2. The sequence of heterologous genes for 3-HP synthesis are listed in Supplementary Table 3.

**Table 1:** Plasmids and strains used in this study

Plasmid			Reference
RLA p603	ZUS1.1-pTEF-RFP turbo-tLip2		This study
RLA p1503	ZLS1.1-pTEF-RFP turbo-tLip2		This study
RLA p1506	ZUS1.1-pTEF-hrGFP-tLip2		This study
RLA P1676	pTDH3-*TcPAND*-tENO1-vLeu2		This study
RLA P1679	pCCW12-*BcBAPAT*-tSSA1- pPGK1-*EcYDFG*-tADH1-vLeu2		This study
RLA P1683	pTDH3-*TcPAND*-tENO1- pCCW12-*BcBAPAT*-tSSA1- pPGK1-*EcYDFG*-tADH1-vLeu2		This study
RLA p1475	ZLS1.1-pTEF-hrGFP-tLip2		This study
RLA p2703	pJET-*PAND*		This study
RLA p2704	pJET-*BAPAT*		This study
RLA p2705	pJET-*YDFG*		This study
RLA p2706	ZUS1.1-pTEF-*PAND*-tLip2		This study
RLA p2707	ZUS1.1-pTEF-*BAPAT*-tLip2		This study
RLA p2708	ZUS1.2-pTEF-*YDFG*-tLip2		This study
RLA p2709	ZLA2.II- pTEF-BAPAT-tLip2-pTEF-*YDFG*-tLip2		This study

*Yarrowia lipolytica*^a^		Abbreviation	Reference
RLA S911	po1d ZLS-pTEF-hrGFP-T3Lip2	*∆ura3*-GFP	This study
RLA S912	po1d ZUS-pTEF-RFPturbo-T3Lip2	*∆leu2*-RFP	This study
RLA S1258	po1d ZLS1.1-pTEF-RFPturbo-TLip2	*∆ura3*-RFP	This study
RLA S1260	po1d ZUS1.1-pTEF-hrGFP-TLip2	*∆leu2*-GFP	This study
RLA S2543	po1d ∆*lys5* ZLS1.1-pTEF-hrGFP-TLip2 + *URA3*	*∆lys5*-GFP	This study
RLA S2544	po1d ∆*lys5* ZUS1.1-pTEF-RFP turbo-TLip2 + *LEU2*	*∆lys5*-RFP	This study
RLA S2547	po1d ∆*trp2* ZLS1.1-pTEF-hrGFP-TLip2 + *URA3*	*∆trp2*-GFP	This study
RLA S2548	po1d ∆*trp2* ZUS1.1-pTEF-RFP turbo-TLip2 + *LEU2*	*∆trp2*-RFP	This study
RLA S2551	po1d ∆*trp4* ZLS1.1-pTEF-hrGFP-TLip2 + *URA3*	*∆trp4*-GFP	This study
RLA S2552	po1d ∆*trp4* ZUS1.1-pTEF-RFP turbo-TLip2 + *LEU2*	*∆trp4*-RFP	This study
RLA S2555	po1d ∆*met5* ZLS1.1-pTEF-hrGFP-TLip2 + *URA3*	*∆met5*-GFP	This study
RLA S2556	po1d ∆*met5* ZUS1.1-pTEF-RFP turbo-TLip2 + *LEU2*	*∆met5*-RFP	This study
RLA S3427	po1d ZUS1.1-pTEF-*PAND*-tLip2 + *LEU2*	*PAND*	This study
RLA S3428	po1d ZLA2.II- pTEF-*BAPAT*-tLip2-pTEF-*YDFG*-tLip2 + *URA3*	*BAPAT*	This study
RLA S3429	po1d ZUS1.1-pTEF-*PAND*-tLip2 + ZLA2.II- pTEF-*BAPAT*-tLip2-pTEF-*YDFG*-tLip2	*PAND-BAPAT*	This study
RLA S3430	po1d ∆*trp2* ZUS1.1-pTEF-*PAND*-tLip2 + *LEU2*	*∆trp2-PAND*	This study
RLA S3431	po1d ∆*trp2* ZLA2.II- pTEF-*BAPAT*-tLip2-pTEF-*YDFG*-tLip2 + *URA3*	*∆trp2-BAPAT*	This study
RLA S3432	po1d ∆*trp2* ZUS1.1-pTEF-*PAND*-tLip2 + ZLA2.II- pTEF-*BAPAT*-tLip2-pTEF-*YDFG*-tLip2	*∆trp2-PAND-BAPAT*	This study
RLA S3433	po1d ∆*trp4* ZUS1.1-pTEF-*PAND*-tLip2 + *LEU2*	*∆trp4-PAND*	This study
RLA S3434	po1d ∆*trp4* ZLA2.II- pTEF-*BAPAT*-tLip2-pTEF-*YDFG*-tLip2 + *URA3*	*∆trp4-BAPAT*	This study
RLA S3435	po1d ∆*trp4* ZUS1.1-pTEF-*PAND*-tLip2 + ZLA2.II- pTEF-*BAPAT*-tLip2-pTEF-*YDFG*-tLip2	*∆trp4-PAND-BAPAT*	This study

*Saccharomyces cerevisiae*			
RLA S335	BY4741 ∆*met5* pTDH3-mScarlet-tADH1-Leu + pHUM	*∆met5*-mScarlet	Aulakh et al. ^10^
RLA S337	BY4741 *Δtrp2* pTDH3-mScarlet-tADH1-Leu + pHUM	*∆trp2*-mScarlet	Aulakh et al. ^10^
RLA S338	BY4741 Δ*trp4* pTDH3-mTagBFP2-tADH1-Leu + pHUM	*∆trp4*-BFP	Aulakh et al. ^10^
RLA S722	BY4741 Δ*lys5* pTDH3-mScarlet-tADH1-Leu + pHUM	*∆lys12*-mScarlet	Aulakh et al. ^10^
RLA S1628	BY4741 pTDH3-*TcPAND*-tENO1+ pHUM	*PAND*	This study
RLA S1629	BY4741 pCCW12-*BcBAPAT*-tSSA1- pPGK1-*EcYDFG*-tADH1-tLeu2 + pHUM + *LEU2*	*BAPAT*	This study
RLA S1585	BY4741 pTDH3-*TcPAND*-tENO1- pCCW12-*BcBAPAT*-tSSA1, pPGK1-*EcYDFG*-tADH1-tLeu2, pHUM + pHUM + *LEU2*	*PAND-BAPAT*	This study
RLA S1604	BY4741*△trp2* + pTDH3-*TcPAND*-tENO1 + pHM + *LYS21*	*∆trp2-PAND*	This study
RLA S1605	BY4741*△trp2* pCCW12-*BcBAPAT*-tSSA1- pPGK1-*EcYDFG*-tADH1-tLeu2 + pHM + *LYS21*	*∆trp2-BAPAT*	This study
RLA S875	BY4741 *∆trp4* + pTDH3-*TcPAND*-tENO1 + pHLM	*∆trp4-PAND*	This study
RLA S894	BY4741 *∆trp4* + pCCW12-*BcBAPAT*-tSSA1- pPGK1-*EcYDFG*-tADH1-tLeu2 + pHLM	*∆trp4-BAPAT*	This study

^a^Gene and species names are written in Italic font.

### Growth and fluorescence analysis of yeast co-culture in 96 well plate

The yeast strains were initially cultured in YPD medium and cultivated overnight at 30 °C and 250 rpm. The cells were then washed three times with distilled water. Subsequently, the cells were inoculated into a 96-well plate containing 200 μl of YNBD media in triplicate. The initial OD_600_ of culture, both monoculture and co-culture, was adjusted to 0.1. The inoculation ratios of the co-culture varied between 10:1, 5:1, 1:1, 1:5, and 1:10. The plate was incubated at 30 °C with continuous shaking for 120 hours. The growth and fluorescence of each strain were monitored using a Spark Tecan or Biotek instrument every 30 min using the following settings: OD_600_, absorbance at 600 nm; GFP, excitation at 485 nm and emission at 535 nm; RFP, excitation at 560 nm and emission at 620 nm; mTAGBFP2, excitation at 400 nm and emission at 465 nm; and mScarlet-I, excitation at 560 nm and emission at 620 nm.

### Population analysis by Flow cytometry

Population of each member in the synthetic community was calculated by the different fluorescence of each strain. Cell fluorescence was measured by an Attune NxT Flow Cytometer (Thermo Scientific) with the following settings: FSC 100 V, SSC 355 V, BL1 345 V, YL2 510 V. Attune Cytometric software was used for data collection. Fluorescence data was collected from at least 10,000 cells for each experiment with three biological replicates.

### Growth analysis of yeast co-culture for 3-HP production in flask

The strains were initially cultured in YNBD medium with tryptophan and cultivated overnight at 30 °C and 250 rpm. The cells were then washed three times with distilled water. Subsequently, the cells were inoculated into the flask containing 10 ml of YNBD media at the initial OD of 0.1, for both monoculture and co-culture. The cells were incubated at 30 °C with 250 rpm for 120 hours. Samples were taken during cultivation to measure the OD_600_ and quantify metabolites in the pathway of 3-HP. OD_600_ values from flask samples were measured by using cuvettes in a UV/Visible spectrophotometer (Biochrom WPA Lightwave II) then normalized by using calibration curve (Supplementary Fig. 17) to compare OD_600_ values between microplate reader and spectrophotometer measured in this study. Raw OD_600_ data from each spectrophotometer are included in Supplementary Table 5.

### Quantification of metabolites

Metabolites including glucose, glycerol, citrate, and ethanol were analyzed by HPLC. The supernatant of cultures at each time point was diluted twenty times with distilled water before the analysis. The HPLC system was equipped with an Thermo Fisher UltiMate 3000 system and Aminex HPX-87H column (300 mm × 7.8 mm, Bio-RAD, USA) coupled to UV (210 nm) and RI detectors. The mobile phase used was 0.01 N H_2_SO_4_ with a flow rate of 0.6 mL/min and the column temperature was T = 35 °C. The raw data from HPLC were processed by Chromeleon software (Thermo Scientific). Concentration of metabolites was quantified by the calibration curve of each standard.

Metabolites in the 3-HP pathway, β-alanine, malonic semialdehyde, and 3-HP, were analyzed by LC-MS. The supernatant of cultures at each time point was diluted four times with 50% acetonitrile for the analysis. The LC-MS system was equipped with an Agilent 1290 Infinity LC system with an Agilent 6550 quadrupole time-of-flight mass spectrometer. An Agilent Poroshell 120 HILIC-Z, 2.1 × 100 mm, 1.9 µm, column was used at a temperature of 45 °C with a solvent flow rate of 0.25 ml min^−1^. LC separation was performed with buffer A (10 mM ammonium formate in water) and buffer B (10 mM ammonium formate in water:ACN 10:90 (vol:vol)). After 0.5 min at 98% buffer B, the composition was changed to 5% buffer B over 2.5 min, then held at 5% buffer B for 1 min. Injection volume was 1 μl, and negative ion spectra were recorded over a mass range of 100–1000 m/z at a rate of 1 spectrum per second. All metabolites were qualified by the functional m/z values. β-Alanine and 3-HP were quantified by the calibration curve of the standards. Malonic semialdehyde was semi-quantified by the standard curves of β-alanine^10^. The raw data from LC-MS were processed by Agilent MassHunter Qualitative Analysis software (Supplementary Table 6).

### Statistics and reproducibility

All experimental data were analyzed using Microsoft Excel 365 and Prism 9.5.0 (GraphPad) software. The error bars in the Figs. 1, 2, and 3 correspond to the standard deviation from *N* = 3 biologically independent samples as described in figure legend. Statistical analyzes were conducted using one-way ANOVA, followed by Bonferroni’s multiple comparisons test with 95% confidence intervals, and *p* values are provided in the source data. The error bars in the Fig. 4 correspond to the standard deviation from *N* = 2 biologically independent samples as described in figure legend.

### Reporting summary

Further information on research design is available in the Nature Portfolio Reporting Summary linked to this article.

## Supplementary information

Supplementary Information

Reporting Summary

Transparent Peer Review file

## Source data

Source Data file_Supplementary Fig.

Source Data file_Figure.zip

## Data Availability

All data generated or analyzed in this study are included in the manuscript and its supplementary information. Source data are provided with this paper.

## References

[1] Tsoi, R., Dai, Z. & You, L. Emerging strategies for engineering microbial communities. *Biotechnol. Adv*. **37**, 107372 (2019). 10.1016/j.biotechadv.2019.03.011 PMC6710121 30880142

[2] McCarty, N. S. & Ledesma-Amaro, R. Synthetic biology tools to engineer microbial communities for biotechnology. *Trends Biotechnol.* **37**, 181–197 (2019). 30497870 10.1016/j.tibtech.2018.11.002 PMC6340809

[3] Bittihn, P., Din, M. O., Tsimring, L. S. & Hasty, J. Rational engineering of synthetic microbial systems: from single cells to consortia. *Curr. Opin. Microbiol.* **45**, 92–99 (2018). 29574330 10.1016/j.mib.2018.02.009 PMC6151159

[4] Darvishi, F., Rafatiyan, S., Abbaspour Motlagh Moghaddam, M. H., Atkinson, E. & Ledesma-Amaro, R. Applications of synthetic yeast consortia for the production of native and non-native chemicals. *Crit. Rev. Biotechnol.* **0**, 1–16 (2022). 10.1080/07388551.2022.2118569 36130800

[5] Bernstein, H. C. & Carlson, R. P. Microbial consortia engineering for cellular factories: In vitro to in silico systems. *Comput. Struct. Biotechnol. J.* **3**, e201210017 (2012). 24688677 10.5936/csbj.201210017 PMC3962199

[6] Shong, J., Jimenez Diaz, M. R. & Collins, C. H. Towards synthetic microbial consortia for bioprocessing. *Curr. Opin. Biotechnol.* **23**, 798–802 (2012). 22387100 10.1016/j.copbio.2012.02.001

[7] Mee, M. T., Collins, J. J., Church, G. M. & Wang, H. H. Syntrophic exchange in synthetic microbial communities. *Proc. Natl. Acad. Sci. USA*. **111**, E2149–56 (2014). 10.1073/pnas.1405641111 PMC4034247 24778240

[8] Shou, W., Ram, S. & Vilar, J. M. G. Synthetic cooperation in engineered yeast populations. *Proc. Natl Acad. Sci. USA.* **104**, 1877–1882 (2007). 17267602 10.1073/pnas.0610575104 PMC1794266

[9] Peng, H. et al. A molecular toolkit of cross-feeding strains for engineering synthetic yeast communities. 10.1038/s41564-023-01596-4 (2023). 10.1038/s41564-023-01596-4 PMC10914607 38326570

[10] Aulakh, S. K. et al. Spontaneously established syntrophic yeast communities improve bioproduction. *Nat. Chem. Biol.* **19**, 951–961 (2023). 37248413 10.1038/s41589-023-01341-2 PMC10374442

[11] Abdel-Mawgoud, A. M. et al. Metabolic engineering in the host Yarrowia lipolytica. *Metab. Eng.* **50**, 192–208 (2018). 30056205 10.1016/j.ymben.2018.07.016

[12] Park, Y. K. & Ledesma-Amaro, R. What makes Yarrowia lipolytica well suited for industry? *Trends Biotechnol.* **41**, 242–254 (2023). 35940976 10.1016/j.tibtech.2022.07.006

[13] Theerachat, M., Tanapong, P. & Chulalaksananukul, W. The culture or co-culture of candida rugosa and yarrowia lipolytica strain rM-4A, or incubation with their crude extracellular lipase and laccase preparations, for the biodegradation of palm oil mill wastewater. *Int. Biodeterior. Biodegrad.* **121**, 11–18 (2017).

[14] Hosseini, F., Hadian, M., Lashani, E. & Moghimi, H. Simultaneous bioreduction of tellurite and selenite by Yarrowia lipolytica, Trichosporon cutaneum, and their co-culture along with characterization of biosynthesized Te–Se nanoparticles. *Microb. Cell Fact.* **22**, 1–13 (2023). 37749532 10.1186/s12934-023-02204-0 PMC10519092

[15] Zhong, Y. et al. Enhanced nitrogen removal via Yarrowia lipolytica-mediated nitrogen and related metabolism of Chlorella pyrenoidosa from wastewater. *Front. Bioeng. Biotechnol.* **11**, 1–12 (2023). 10.3389/fbioe.2023.1159297 PMC10325826 37425353

[16] Koutinas, M., Patsalou, M., Stavrinou, S. & Vyrides, I. High temperature alcoholic fermentation of orange peel by the newly isolated thermotolerant Pichia kudriavzevii KVMP10. *Lett. Appl. Microbiol.* **62**, 75–83 (2016). 26510181 10.1111/lam.12514

[17] Liu, X. et al. One-pot fermentation for erythritol production from distillers grains by the co-cultivation of Yarrowia lipolytica and Trichoderma reesei. *Bioresour. Technol.* **351**, 127053 (2022). 35337991 10.1016/j.biortech.2022.127053

[18] Liu, X., Lv, J., Zhang, T. & Deng, Y. Direct Conversion of Pretreated Straw Cellulose into Citric Acid by Co-cultures of Yarrowia lipolytica SWJ-1b and Immobilized Trichoderma reesei Mycelium. *Appl. Biochem. Biotechnol.* **173**, 501–509 (2014). 24659047 10.1007/s12010-014-0856-8 PMC4007025

[19] Bai, S. et al. Mutualistic microbial community of Bacillus amyloliquefaciens and recombinant Yarrowia lipolytica co-produced lipopeptides and fatty acids from food waste. *Chemosphere* **310**, 136864 (2023). 36243085 10.1016/j.chemosphere.2022.136864

[20] Ariana, M. & Hamedi, J. Enhanced production of nisin by co-culture of Lactococcus lactis sub sp. lactis and Yarrowia lipolytica in molasses based medium. *J. Biotechnol.* **256**, 21–26 (2017). 28694185 10.1016/j.jbiotec.2017.07.009

[21] Marsafari, M., Azi, F., Dou, S. & Xu, P. Modular co-culture engineering of Yarrowia lipolytica for amorphadiene biosynthesis. *Microb. Cell Fact.* **21**, 1–10 (2022). 36587216 10.1186/s12934-022-02010-0 PMC9805133

[22] Liu, S. et al. Engineering 3-Hydroxypropionic Acid Production from Glucose in Yarrowia lipolytica through Malonyl-CoA Pathway. *J. Fungi* **9**, 573 (2023). 10.3390/jof9050573 PMC10219321 37233284

[23] Borodina, I. et al. Establishing a synthetic pathway for high-level production of 3-hydroxypropionic acid in Saccharomyces cerevisiae via β-alanine. *Metab. Eng.* **27**, 57–64 (2015). 25447643 10.1016/j.ymben.2014.10.003

[24] Zengler, K. & Zaramela, L. S. The social network of microorganisms - How auxotrophies shape complex communities. *Nat. Rev. Microbiol.* **16**, 383–390 (2018). 29599459 10.1038/s41579-018-0004-5 PMC6059367

[25] Zhang, H. & Wang, X. Modular co-culture engineering, a new approach for metabolic engineering. *Metab. Eng.* **37**, 114–121 (2016). 27242132 10.1016/j.ymben.2016.05.007

[26] Brenner, K., You, L. & Arnold, F. H. Engineering microbial consortia: a new frontier in synthetic biology. *Trends Biotechnol.* **26**, 483–489 (2008). 18675483 10.1016/j.tibtech.2008.05.004

[27] Kaleta, C., Schäuble, S., Rinas, U. & Schuster, S. Metabolic costs of amino acid and protein production in Escherichia coli. *Biotechnol. J.* **8**, 1105–1114 (2013). 23744758 10.1002/biot.201200267

[28] Raiford, D. W. et al. Do amino acid biosynthetic costs constrain protein evolution in Saccharomyces cerevisiae? *J. Mol. Evol.* **67**, 621–630 (2008). 18937004 10.1007/s00239-008-9162-9

[29] Barton, M. D., Delneri, D., Oliver, S. G., Rattray, M. & Bergman, C. M. Evolutionary systems biology of amino acid biosynthetic cost in yeast. *PLoS One* **5**, e11935 (2010). 10.1371/journal.pone.0011935 PMC2923148 20808905

[30] Campbell, K. et al. Self-establishing communities enable cooperative metabolite exchange in a eukaryote. *Elife* **4**, 1–23 (2015). 10.7554/eLife.09943 PMC4695387 26499891

[31] Kerner, A., Park, J., Williams, A. & Lin, X. N. A programmable Escherichia coli consortium via tunable symbiosis. *PLoS One* **7**, e34032 (2012). 22479509 10.1371/journal.pone.0034032 PMC3316586

[32] Lubbe, A., Bowen, B. P. & Northen, T. Exometabolomic analysis of cross-feeding metabolites. *Metabolites* **7**, 50 (2017). 10.3390/metabo7040050 PMC5746730 28976938

[33] Chodkowski, J. L. & Shade, A. Exometabolite dynamics over stationary phase reveal strain-specific responses. *mSystems* **5**, 50 (2020). 10.1128/mSystems.00493-20 PMC7762789 33361318

[34] Zhou, K., Qiao, K., Edgar, S. & Stephanopoulos, G. Distributing a metabolic pathway among a microbial consortium enhances production of natural products. *Nat. Biotechnol.* **33**, 377–383 (2015). 25558867 10.1038/nbt.3095 PMC4867547

[35] Yuzbashev, T. V. et al. A DNA assembly toolkit to unlock the CRISPR/Cas9 potential for metabolic engineering. *Commun. Biol*. **6**, 858 (2023). 10.1038/s42003-023-05202-5 PMC10439232 37596335

