# Supplementary material for: Engineered cross-feeding creates inter- and intra-species synthetic yeast communities with enhanced bioproduction
Source: Nat Commun. 2024 Oct 16;15:8924. doi: 10.1038/s41467-024-53117-4 (PMC11484764; doi:10.1038/s41467-024-53117-4)

Engineered cross-feeding creates inter- and intra-species synthetic yeasts communities with enhanced bioproduction

Supplementary Tables

Supplementary Table 1. Growth rate and maximal OD from syntrophic cocultures of *Y. lipolytica* auxotroph strains (inoculation ratio = 1:1).

Supplementary Table 2. Primer sequences used in this study.

Supplementary Table 3. Gene sequences for 3-HP production used in this study.

Supplementary Table 4. Production of metabolites in the 3-HP biosynthetic pathway from the intra- and interspecies synthetic communities.

Supplementary Table 5. Raw OD_{600} data and normalized OD_{600} data of Figure 4.

Supplementary Table 6. Raw data of metabolite analysis by LC/MS.

Supplementary Figures

Supplementary Figure 1. Growth profile of syntrophic coculture of *Y. lipolytica* auxotroph strains (inoculation ratio = 1:1).

Supplementary Figure 2. Growth profile of syntrophic coculture of *Y. lipolytica* auxotroph strains with a specific auxotroph (inoculation ratio = 1:1).

Supplementary Figure 3. Glucose consumption of syntrophic coculture of *Y. lipolytica* auxotroph strains (inoculation ratio = 1:1)

Supplementary Figure 4. Glucose consumption of selected syntrophic coculture of *Y. lipolytica* auxotroph strains at different inoculation ratio from 10:1 to 1:10.

Supplementary Figure 5. Population of co-culture between YL*Δura3* and YL*Δtrp4* at different inoculation ratios.

Supplementary Figure 6. Population of co-culture between YL*Δmet5* and YL*Δtrp4* at different inoculation ratios.

Supplementary Figure 7. Population of co-culture between YL*Δtrp2* and YL*Δtrp4* at different inoculation ratios.

Supplementary Figure 8. Growth profile of interspecies syntrophic coculture between *Y. lipolytica* and *S. cerevisiae* (inoculation ratio = 1:1).

Supplementary Figure 9. Population of co-culture among YL*Δtrp2*, YL*Δtrp4*, SC*Δtrp2*, and SC*Δtrp4* at different inoculation ratios.

Supplementary Figure 10. Profiles of growth and metabolites co-culture among YL*Δtrp2*, YL*Δtrp4*, SC*Δtrp2*, and SC*Δtrp4* at different inoculation ratios in aerobic condition.

Supplementary Figure 11. Profiles of growth and metabolites co-culture among YL*Δtrp2*, YL*Δtrp4*, SC*Δtrp2*, and SC*Δtrp4* at different inoculation ratios in different culture condition.

Supplementary Figure 12. Division of labor in syntrophic community of *Y. lipolytica* for bioproduction of 3-hydroxypropionic acid.

Supplementary Figure 13. Division of labor in interspecies syntrophic community of *Y. lipolytica* and *S. cerevisiae* for bioproduction of 3-hydroxypropionic acid.

Supplementary Figure 14. Division of labor in syntrophic community of *S. cerevisiae* for bioproduction of 3-hydroxypropionic acid.

Supplementary Figure 15. Profiles of glucose consumption and byproduct formation in the co-cultures for 3-HP production.

Supplementary Figure 16. (a) Metabolic pathway including synthetic 3-HP pathway. (b) Production of metabolites (citrate, pyruvate, β-alanine, malonic semialdehyde, and 3-hydroxypropionic acid) from mono- and co-cultures.

Supplementary Figure 17. The calibration curve of OD_{600} between microplate reader and spectrophotometer. OD600 from spectrophotometer was calibrated to the value of microplate reader in figures in the main manuscript and Supplementary Figures.

Supplementary Figure 18. Comparison of division of labor in the *S. cerevisiae* synthetic community between previous study and this study. Supplementary Data 8 of previous study (Aulakh et al. 2013) was used.

Supplementary Table 1. Growth rate and maximal OD from syntrophic cocultures of *Y. lipolytica* auxotroph strains (inoculation ratio = 1:1). Values represent average and standard deviation (n=3).

Auxotrophic pair	maximal growth rate (h^{-1})		maximal OD	
Δura3-Δleu2	0.035 ± 0.011	+	0.244 ± 0.010	
Δura3-Δlys5	0.066 ± 0.019	++	0.329 ± 0.009	+
Δura3-Δtrp2	0.032 ± 0.005	+	0.360 ± 0.019	+
Δura3-Δtrp4	0.072 ± 0.023	++	0.700 ± 0.025	++
Δura3-Δmet5	0.047 ± 0.003	+	0.646 ± 0.055	++
Δleu2-Δlys5	0.016 ± 0.006		0.221 ± 0.013	
Δleu2-Δtrp2	0.035 ± 0.007	+	0.332 ± 0.005	+
Δleu2-Δtrp4	0.048 ± 0.010	+	0.609 ± 0.038	++
Δleu2-Δmet5	0.011 ± 0.007		0.262 ± 0.023	
Δlys5-Δtrp2	0.031 ± 0.005	+	0.359 ± 0.041	+
Δlys5-Δtrp4	0.040 ± 0.008	+	0.566 ± 0.043	++
Δlys5-Δmet5	0.013 ± 0.002		0.393 ± 0.047	+
Δtrp2-Δtrp4	0.084 ± 0.003	++	0.513 ± 0.041	+
Δtrp2-Δmet5	0.019 ± 0.006		0.304 ± 0.041	
Δtrp4-Δmet5	0.029 ± 0.006	+	0.627 ± 0.063	++

growth rate		OD	
$\mu_{max} \geqq 0.06$	++	OD ≧ 0.55	++
$0.02 \leqq \mu_{max} < 0.06$	+	0.32 ≦ OD < 0.55	+
$\mu_{max} \leqq 0.02$		OD ≦ 0.32	

Supplementary Table 2. Primer sequences used in this study.

Primer	Sequence (5' - 3')
pTEF-internal-Fw	CCATGCCGGACGCAAAATAGACTAC
Tlip2-internal-Rev	CGATTTGTCTTAGAGGAACGCATATACAGTAATC
hrGFP-internal-Rev	GCTGTAGAACTTGCCGCTGTTCAG
mRFP1-internal-Rev	CGGTAACAACACCACCGTCTTCG
URA3-500-Rev	CTGGCCAGCTTCTCGTTGGG
URA3-ATG-Fw	ATGCCCTCCTACGAAGCTCGAG
LEU2-800-Rev	GGCCTCGTCGGAGATGATATCGC
LEU2-ATG-Fw	ATGGAACCCGAAACTAAGAAGACCAAGAC
P-LYS5-Fw	CCCATCACCACGGATTACTTAGGTGG
P-LYS5-Rev	CGTGCATTACCCTGTTATCCCTAGCCTGGTTGAAGTCGTTCTGGG
T-LYS5-Fw	GGCTAGGGATAACAGGGTAATGCACGTCTAGAATTTGAACCTATCG
T-LYS5-Rev	GGCCAGAAATTTCTGGTACGACATG
Ex-LYS5-Fw	CGACATTCAGATGATTTCTCAGGTGCC
Ex-LYS5-Rev	CCCGTTTGAACAGTACACTTGGC
P MET5-Fw	ATAAGAATGCGGCCGCGGTACAAGTACCAAGCCAAGG
P MET5-Rev	CGATTACCCTGTTATCCCTACCGTTGCAGTCCTCCATTGAGG
T MET5-Fw	GGTAGGGATAACAGGGTAATCGGTCATCAAGGAGACCACTGC
T MET5-Rev	ATAGTTTAGCGGCCGCCACGATCATGTGACCAGACC
Ex-MET5-Fw	CGGTGCTGTACCTGTACAGTAC
Ex-MET5-Rev	GCTTCTAGCAGGTTCACTTGG
P TRP2-Fw	ATAAGAATGCGGCCGCGAGCTGATGTGGCAAGGTC
P TRP2-Rev	CGATTACCCTGTTATCCCTACCCGAAATGTATCCGATGGCTCC
T TRP2-Fw	GGTAGGGATAACAGGGTAATCGGAGGCGATGAAGATGCACTTC
T TRP2-Rev	ATAGTTTAGCGGCCGCCAAGTATGGCTGTCTCGGAAG
P TRP4-Fw	ATAAGAATGCGGCCGCGCACTTGGTCAGGTATAGCG
P TRP4-Rev	CGATTACCCTGTTATCCCTACCGTGTTGAGAGGCAGAGACATG
T TRP4-Fw	GGTAGGGATAACAGGGTAATCGGAAGGAGGCATAAACGCTCG
T TRP4-Rev	ATAGTTTAGCGGCCGCGAGTCGGAGTTGTGTTCAACC
GG-BAPAT-Fw	GCATCGTCTCATCGGGGTCTCAAATGGAATTAATGATCGTTCAAGTAACTGAGC
BAPAT-600-Rev	GGATTTCTGTAGGTATCAGGTGGG
GG-BAPAT-Rev	CTGACGTCTCAGGTCGGTCTCATAGATCAGGATCCTAGTTGAGCAAGACATTCC
GG-YDFG-Fw	GCATCGTCTCATCGGGGTCTCAAATGATAGTCTTGGTCACAGGTGCAAC
YDFG-700-Rev	CGGGCATCATTTCCAAGG
GG-YDFG-Rev	CTGACGTCTCAGGTCGGTCTCATAGATCAGGATCCTTGCCTATGCACG
GG-PAND-Fw	GCATCGTCTCATCGGGGTCTCAAATGCCTGCAACAGGAGAGGAC
PAND-500-Rev	GGGTTCAAAGCGTCAGTTGCC
GG-PAND-Rev	CTGACGTCTCAGGTCGGTCTCATAGATCAGGATCCTAAATCAGAACCTAGACG

Supplementary Table 3. Gene sequences for 3-HP production used in this study.

Gene	Sequence
PAND	ATGCCTGCAACAGGAGAGGACCAAGACCTAGTTCAAGACTTAATAGAGGAACCAGCAACATTTAGTGATG<br>CCGTATTATCCAGCGACGAGGAGCTATTTCATCAGAAGTGCCCTAAGCCTGCTCCTATTTATAGCCCGGTGT<br>CTAAACCCGTGTCCTTTGAATCATTGCCTAACCGTAGGTTGCACGAGGAGTTTCTAAGGTCTTCTGTGGACG<br>TTTTATTACAGGAAGCTGTTTTCGAAGGTACGAACAGGAAGAACAGAGTCCTTCAGTGGAGAGAGCCAGA<br>GGAGCTAAGAAGGTTGATGGACTTCGGAGTCAGGAGTGCCCCTTCTACCCACGAGGAGTTACTAGAGGTT<br>TTAAAGAAAGTAGTAACCTATAGTGTTAAAACAGGACACCCATATTTCGTTAACCAACTATTTAGCGCGGTT<br>GACCCTTATGGATTAGTTGCACAATGGGCAACTGACGCTTTGAACCCTAGTGTATATACTTACGAGGTGAG<br>TCCCGTATTTGTATTGATGGAAGAGGTCGTCCTTAGGGAGATGAGGGCAATCGTCGGATTTGAGGGCGGT<br>AAAGGGGACGGTATCTTCTGTCCTGGCGGGTCAATAGCCAATGGCTACGCTATCTCCTGTGCAAGATATCG<br>TTTTATGCCTGACATCAAGAAGAAGGGGTTGCATTCCTTGCCAAGATTAGTATTGTTCACATCTGAGGATGC<br>GCACTATTCCATAAAGAAACTTGCATCTTTCCAGGGCATTGGGACCGACAACGTATATTTGATACGTACCG<br>ACGCAAGAGGCAGGATGGACGTTTCACACTTAGTTGAAGAGATAGAGCGTAGCTTGAGGGAAGGGGCAG<br>CACCTTTCATGGTTTCGGCTACCGCAGGGACCACTGTTATCGGAGCTTTCGATCCTATAGAAAAGATCGCC<br>GATGTCTGCCAAAAGTACAAGCTTTGGCTACATGTAGACGCTGCGTGGGGTGGCGGGGCATTGGTCAGCG<br>CGAAGCACCGTCATCTACTGAAAGGAATTGAGAGGGCTGATTCAGTTACATGGAACCCTCATAAGCTATTA<br>ACCGCCCCACAGCAGTGCTCAACTTTATTATTGAGACATGAGGGTGTTCTAGCTGAAGCACATTCCACTAAT<br>GCCGCTTACCTGTTCCAGAAGGACAAGTTTTATGATACTAAGTACGACACAGGAGACAAACACATTCAGTG<br>TGGAAGGAGAGCAGACGTTCTAAAATTCTGGTTCATGTGGAAAGCAAAGGGCACAAGCGGGCTTGAGAA<br>ACACGTGGACAAAGTTTTCGAGAACGCCAGGTTCTTTACAGATTGTATTAAGAACCGTGAGGGCTTTGAGA<br>TGGTAATTGCAGAGCCAGAATACACAAACATATGTTTCTGGTATGTCCCAAAGTCTCTACGTGGGCGTAAA<br>GACGAGGCCGACTATAAAGACAAACTACACAAAGTCGCTCCAAGAATCAAGGAGCGTATGATGAAAGAG<br>GGCTCTATGATGGTAACCTATCAGGCACAGAAGGGTCACCCTAACTTCTTTCGTATAGTCTTTCAAAACTCT<br>GGACTAGACAAAGCCGACATGGTACATTTGGTCGAAGAGATTGAGCGTCTAGGTTCTGATTTAGGATCCT<br>GA
BAPAT	ATGGAATTAATGATCGTTCAAGTAACTGAGCAAACACAGAGCTTAAAGAAGACCGACGAGAAATATTTAT<br>GGCATGCCATGAGGGGCGCAGCGCCATCTCCGACTAATTTGATCATTACCAAAGCAGAGGGTGCGTGGGT<br>CACCGATATTGATGGTAATCGTTATCTAGATGGCATGTCTGGCTTGTGGTGTGTTAACGTCGGCTATGGGA<br>GAAAAGAACTAGCAAGGGCTGCATTCGAACAACTTGAAGAAATGCCTTACTTCCCTTTAACTCAGTCTCAT<br>GTACCTGCAATAAAGCTAGCAGAGAAATTGAATGAGTGGCTGGATGACGAATACGTCATTTTCTTCTCCAA<br>CTCTGGGTCGGAAGCTAATGAAACGGCATTCAAAATAGCAAGACAATATCACCAACAGAAAGGTGACCAC<br>GGTAGATATAAGTTTATTTCAAGATATCGTGCGTATCATGGAAATTCCATGGGTGCCCTGGCTGCAACTGG<br>TCAAGCCCAAAGAAAATACAAGTACGAGCCACTAGGGCAAGGATTCCTACACGTAGCCCCACCTGATACCT<br>ACAGAAATCCAGAAGATGTCCATACCCTGGCATCAGCTGAGGAAATCGATCGTGTTATGACGTGGGAATT<br>GTCTCAAACCGTGGCTGGAGTGATCATGGAACCCATCATTACGGGTGGTGGTATTCTGATGCCACCTGATG<br>GATATATGGAAAAGGTAAAGGAAATTTGCGAAAAGCACGGAGCACTTTTAATCTGCGATGAGGTTATCTG<br>CGGATTTGGCCGTACTGGGAAACCGTTTGGGTTCATGAACTATGGTGTCAAGCCGGACATTATTACTATGG<br>CGAAAGGTATCACCAGCGCATACCTACCTCTTTCTGCCACAGCTGTGCGTCGTGAAGTGTACGAGGCTTTT<br>GTAGGTTCCGACGATTACGACAGATTTAGACATGTAAACACCTTTGGTGGAAATCCAGCAGCCTGTGCATT<br>GGCTTTAAAGAACCTGGAAATAATGGAAAACGAAAAGCTGATTGAAAGATCAAAGGAACTGGGTGAAAG<br>ATTGTTGTACGAATTAGAGGATGTGAAGGAACATCCCAATGTGGGTGATGTAAGGGGCAAAGGTTTGTTG<br>CTGGGAATCGAACTAGTTGAGGATAAACAAACTAAGGAACCTGCATCTATAGAAAAGATGAATAAGGTTA<br>TTAATGCATGCAAGGAGAAAGGACTGATCATCGGGAAGAATGGGGATACAGTAGCTGGGTATAACAATAT<br>CCTACAACTGGCGCCCCCGTTGTCAATCACCGAAGAGGACTTCACTTTTATAGTTAAAACGATGAAGGAAT<br>GTCTTGCTCAACTAGGATCCTGA
YDFG	ATGATAGTCTTGGTCACAGGTGCAACGGCGGGTTTCGGTGAATGTATTACTCGTAGGTTCATACAACAGGG<br>GCATAAGGTTATAGCGACGGGAAGGAGACAGGAAAGATTGCAAGAATTAAAAGACGAACTTGGCGATAA<br>TTTGTATATTGCTCAATTGGATGTACGTAACAGGGCAGCTATAGAAGAAATGTTAGCCTCATTGCCAGCAG<br>AGTGGTGTAACATAGACATTCTAGTAAACAATGCCGGTCTTGCGTTAGGAATGGAACCAGCGCACAAGGC<br>CTCTGTTGAAGATTGGGAGACTATGATCGATACGAACAATAAGGGTTTGGTCTATATGACGCGTGCCGTCT<br>TGCCGGGTATGGTGGAAAGAAACCATGGCCACATAATTAATATTGGGTCCACAGCGGGTTCATGGCCATA<br>CGCTGGCGGGAACGTGTACGGTGCTACTAAAGCTTTTGTAAGGCAATTTAGCCTGAATTTGAGGACCGATT<br>TGCATGGCACTGCCGTTAGAGTAACAGACATTGAGCCCGGTCTTGTTGGCGGAACCGAATTTAGTAACGTG<br>AGGTTTAAGGGTGACGATGGTAAGGCAGAGAAAACTTATCAAAATACGGTAGCCTTGACACCAGAAGATG<br>TGTCAGAGGCTGTATGGTGGGTATCAACTTTACCGGCGCATGTAAATATAAATACCTTGGAAATGATGCCC<br>GTCACACAATCCTACGCAGGCCTTAACGTGCATAGGCAAGGATCCTGA

Supplementary Table 4. Production of metabolites in the 3-HP biosynthetic pathway from the intra- and interspecies synthetic communities. Values represent average and standard deviation (n=2).

Strain(s)	Inoculation ratio	β-Alanine (mM)	MSA (mM)	3-HP (mM)
SC mono (P+B)		0.194 ± 0.0259	0.051 ± 0.0085	0.112 ± 0.0175
YL mono (P+B)		0.071 ± 0.0007	0.021 ± 0.0017	0.242 ± 0.0177
SC WT-P/SC WT-B	1:1	0.190 ± 0.0021	0.061 ± 0.0029	0.065 ± 0.0047
YL WT-P/YL WT-B	1:1	0.092 ± 0.0007	0.019 ± 0.0005	0.113 ± 0.0264
SC WT-B/YL WT-P	1:1	0.051 ± 0.0027	0.038 ± 0.0042	nd
SC △trp2-B/SC △trp4-P	10:1	nd	1.599 ± 0.0267	0.104 ± 0.0316
	1:1	nd	0.058 ± 0.0142	0.14 ± 0.0317
	1:10	nd	0.066 ± 0.0066	nd
YL △trp2-B/YL △trp4-P	10:1	0.02 ± 0.0196	1.089 ± 0.0167	4.671 ± 0.0635
	1:1	0.261 ± 0.0347	1.761 ± 0.0910	2.666 ± 0.0473
	1:10	0.103 ± 0.0026	0.039 ± 0.0009	0.265 ± 0.0355
SC △trp2-B/YL △trp4-P	10:1	nd	1.466 ± 0.0637	4.500 ± 0.0119
	1:1	nd	0.159 ± 0.0210	0.204 ± 0.0008
	1:10	0.088 ± 0.0014	1.453 ± 0.0029	0.076 ± 0.0150
YL △trp2-B/SC △trp4-P	10:1	0.163 ± 0.0030	1.473 ± 0.0236	3.962 ± 0.0009
	1:1	0 .182 ± 0.0032	0.163 ± 0.0613	0.706 ± 0.1052
	1:10	0.201 ± 0.0213	0.950 ± 0.1025	1.119 ± 0.0477

*nd: not detected

Supplementary Table 5. Raw OD_{600} data and normalized OD_{600} data of Figure 4.

a) OD_{600} data from the spectrophotometer

Strain	Inoculation ratio		Time (hr)					
			0	24	48	72	96	120
SC mono (P+B)		rep 1	0.1	14.1	22.7	27.7	30.7	37.5
		rep 2	0.1	13.7	23.2	27.2	28.2	37.6
YL mono (P+B)		rep 1	0.1	42.4	53.8	52.2	59.3	58.2
		rep 2	0.1	41.4	54.9	56.2	57.9	61.8
SC WT-P/SC WT-B	1:1	rep 1	0.1	21.7	28.9	35.2	34.6	42
		rep 2	0.1	23.6	30	36.4	38.9	43.7
YL WT-P/YL WT-B	1:1	rep 1	0.1	39.1	49.9	52.3	53.7	57.9
		rep 2	0.1	36	43.6	49.1	46.5	49.4
SC WT-B/YL WT-P	1:1	rep 1	0.1	28.7	42.3	37.4	30.5	43.5
		rep 2	0.1	33	45.3	40.5	42	44
SC △trp2-B/SC △trp4-P	1:10	rep 1	0.1	0.96	16.5	20.3	25.4	31.3
		rep 2	0.1	1.28	16.6	22.7	28.8	33.9
	1:1	rep 1	0.1	8.94	43.1	32.6	36	39.4
		rep 2	0.1	9.04	43.7	33.4	35.8	41.3
	10:1	rep 1	0.1	2.58	4.3	11.74	15.12	19.3
		rep 2	0.1	2.68	4.14	11.54	15.4	20.4
YL △trp2-B/YL △trp4-P	1:10	rep 1	0.1	2.54	46	49.9	51.8	52.2
		rep 2	0.1	1.66	51.8	51.3	64.2	58
	1:1	rep 1	0.1	4.34	26.5	21.6	21.4	29.1
		rep 2	0.1	4.74	31.3	24.7	22.4	28.7
	10:1	rep 1	0.1	0.82	2.62	5.78	7.74	8.96
		rep 2	0.1	0.92	3.16	6.02	7.94	9.5
SC △trp2-B/YL △trp4-P	1:10	rep 1	0.1	0.66	10.6	22.3	24.1	25.7
		rep 2	0.1	0.98	9.9	26.2	25.4	24
	1:1	rep 1	0.1	7.98	39.7	34.3	41.1	46.3
		rep 2	0.1	8.02	39.1	32.4	38.5	42.4
	10:1	rep 1	0.1	2.48	2.38	3.06	3.9	4.6
		rep 2	0.1	2.76	2.24	3.64	4.38	5.2
YL △trp2-B/SC △trp4-P	1:10	rep 1	0.1	0.34	16.4	39.3	49.3	44.6
		rep 2	0.1	0.64	16.3	42.2	52	53.1
	1:1	rep 1	0.1	3.3	52.8	51.2	47.8	56.9
		rep 2	0.1	3.44	53.1	54.8	48.9	58.9
	10:1	rep 1	0.1	0.84	4.22	13.08	18.58	27.3
		rep 2	0.1	0.1	4.2	12.46	19.5	28.2

(b) Normalized OD_{600} data

Strain	Inoculation ratio		Time (hr)					
			0	24	48	72	96	120
SC mono (P+B)		rep 1	0.06	0.87	1.37	1.66	1.83	2.22
		rep 2	0.06	0.85	1.40	1.63	1.69	2.23
YL mono (P+B)		rep 1	0.06	2.51	3.17	3.07	3.49	3.42
		rep 2	0.06	2.45	3.23	3.31	3.40	3.63
SC WT-P/SC WT-B	1:1	rep 1	0.06	1.31	1.73	2.09	2.06	2.48
		rep 2	0.06	1.42	1.79	2.16	2.31	2.58
YL WT-P/YL WT-B	1:1	rep 1	0.06	2.32	2.94	3.08	3.16	3.40
		rep 2	0.06	2.14	2.58	2.90	2.74	2.91
SC WT-B/YL WT-P	1:1	rep 1	0.06	1.72	2.50	2.22	1.82	2.57
		rep 2	0.06	1.96	2.68	2.40	2.48	2.60
SC △trp2-B/SC △trp4-P	1:10	rep 1	0.06	0.11	1.01	1.23	1.52	1.87
		rep 2	0.06	0.13	1.02	1.37	1.72	2.02
	1:1	rep 1	0.06	0.57	2.55	1.94	2.14	2.33
		rep 2	0.06	0.58	2.58	1.99	2.13	2.44
	10:1	rep 1	0.06	0.20	0.30	0.73	0.93	1.17
		rep 2	0.06	0.21	0.29	0.72	0.95	1.24
YL △trp2-B/YL △trp4-P	1:10	rep 1	0.06	0.20	2.72	2.94	3.05	3.07
		rep 2	0.06	0.15	3.05	3.02	3.77	3.41
	1:1	rep 1	0.06	0.31	1.59	1.30	1.29	1.74
		rep 2	0.06	0.33	1.87	1.48	1.35	1.72
	10:1	rep 1	0.06	0.10	0.21	0.39	0.50	0.57
		rep 2	0.06	0.11	0.24	0.40	0.51	0.60
SC △trp2-B/YL △trp4-P	1:10	rep 1	0.06	0.09	0.67	1.35	1.45	1.54
		rep 2	0.06	0.11	0.63	1.57	1.52	1.44
	1:1	rep 1	0.06	0.52	2.35	2.04	2.43	2.73
		rep 2	0.06	0.52	2.32	1.93	2.28	2.51
	10:1	rep 1	0.06	0.20	0.19	0.23	0.28	0.32
		rep 2	0.06	0.21	0.18	0.27	0.31	0.36
YL △trp2-B/SC △trp4-P	1:10	rep 1	0.06	0.07	1.00	2.33	2.91	2.63
		rep 2	0.06	0.09	1.00	2.50	3.06	3.13
	1:1	rep 1	0.06	0.25	3.11	3.02	2.82	3.35
		rep 2	0.06	0.25	3.13	3.22	2.88	3.46
	10:1	rep 1	0.06	0.10	0.30	0.81	1.13	1.63
		rep 2	0.06	0.06	0.30	0.78	1.18	1.69

Supplementary Table 6. Raw data of metabolite analysis by LC/MS.

Strain	Inoculation ratio		β-Alanine		MSA		3-HP	
			Retention time (min)	Peak area	Retention time (min)	Peak area	Retention time (min)	Peak area
SC mono (P+B)	Mono culture	rep 1	2.915	9036	2.598	6856	2.982	4859
		rep 2	2.909	11823	2.609	4896	2.992	6669
YL mono (P+B)	Mono culture	rep 1	2.842	3875	2.558	2271	2.908	11548
		rep 2	2.853	3803	2.553	2671	2.919	13380
SC WT-P/SC WT-B	1:1	rep 1	2.904	10328	2.587	6710	2.987	3586
		rep 2	2.904	10107	2.587	7386	2.937	3097
YL WT-P/YL WT-B	1:1	rep 1	2.839	4908	2.606	2234	2.906	4474
		rep 2	2.838	4986	2.588	2108	2.905	7200
SC WT-B/YL WT-P	1:1	rep 1	2.835	2908	2.585	3850	nd	nd
		rep 2	2.837	2619	2.57	4831	nd	nd
SC △trp2-B/SC △trp4-P	1:10	rep 1	nd	nd	2.603	6904	nd	nd
		rep 2	nd	nd	2.578	8426	nd	nd
SC △trp2-B/SC △trp4-P	1:1	rep 1	nd	nd	2.571	5103	2.938	8875
		rep 2	nd	nd	2.587	8391	2.92	5600
SC △trp2-B/SC △trp4-P	10:1	rep 1	nd	nd	2.586	181695	2.902	3714
		rep 2	nd	nd	2.583	187868	2.916	6974
YL △trp2-B/YL △trp4-P	1:10	rep 1	2.843	5687	2.577	4372	2.91	11835
		rep 2	2.841	5412	2.574	4584	2.891	15503
YL △trp2-B/YL △trp4-P	1:1	rep 1	2.833	15945	2.583	213951	2.9	135148
		rep 2	2.83	12201	2.597	192932	2.914	140034
YL △trp2-B/YL △trp4-P	10:1	rep 1	nd	nd	2.589	123887	2.906	244342
		rep 2	2.837	2115	2.571	127735	2.904	237788
SC △trp2-B/YL △trp4-P	1:10	rep 1	2.84	4801	2.606	168162	2.89	3147
		rep 2	2.838	4653	2.605	167499	2.939	4699
SC △trp2-B/YL △trp4-P	1:1	rep 1	nd	nd	2.573	15980	2.906	10553
		rep 2	nd	nd	2.584	20822	2.901	10468
SC △trp2-B/YL △trp4-P	10:1	rep 1	nd	nd	2.568	161980	2.901	232860
		rep 2	nd	nd	2.583	176699	2.9	231634
YL △trp2-B/SC △trp4-P	1:10	rep 1	2.839	9685	2.589	121604	2.905	60209
		rep 2	2.839	11981	2.589	97910	2.906	55284
YL △trp2-B/SC △trp4-P	1:1	rep 1	2.836	9653	2.569	25960	2.902	41879
		rep 2	2.833	9997	2.566	11793	2.899	31016
YL △trp2-B/SC △trp4-P	10:1	rep 1	2.836	8613	2.586	167435	2.903	204517
		rep 2	2.833	8941	2.583	172882	2.9	204426

*nd: not detected

Supplementary Figure 1. Growth profile of syntrophic coculture of *Y. lipolytica* auxotroph strains (inoculation ratio = 1:1). Values represent averages and error bars denote standard deviation (n=3).

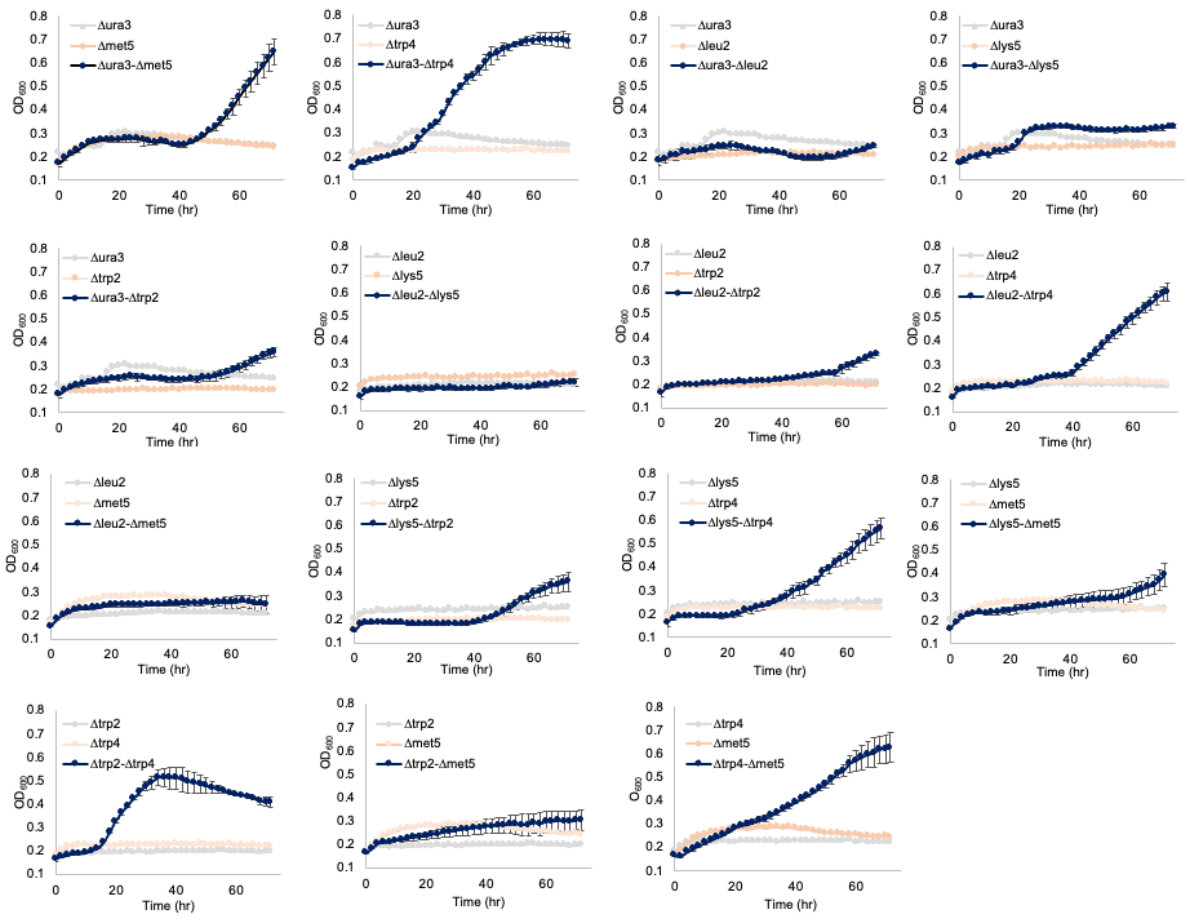

Supplementary Figure 2. Growth profile of syntrophic coculture of *Y. lipolytica* auxotroph strains with a specific auxotroph (inoculation ratio = 1:1). (a) coculture pairs with *Δura3*, (b) coculture pairs with *Δleu2*, (c) coculture pairs with *Δlys5*, (d) coculture pairs with *Δtrp2*, (e) coculture pairs with *Δtrp4*, (f) coculture pairs with *Δmet5*. Values represent averages and error bars denote standard deviation(n=3).

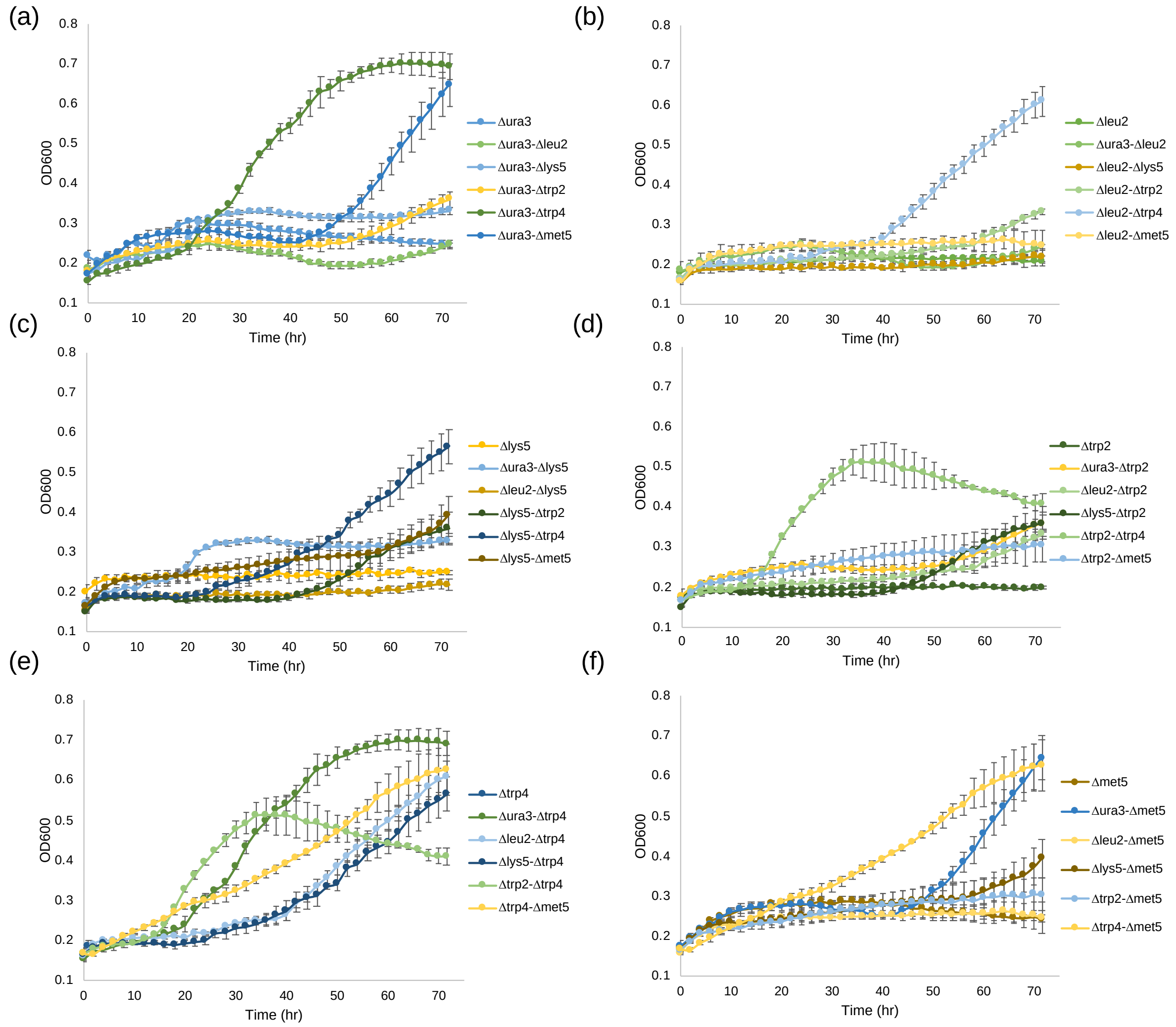

Supplementary Figure 3. Glucose consumption of monocultures (a single auxotroph) and cocultures (a pair of two auxotroph with 1:1 inoculation ratio) at 72 hours of cultivation. Values represent averages and error bars denote standard deviation (n=3). One-way ANOVA, followed by Bonferroni's multiple comparisons test with 95% confidence intervals were performed using GraphPad Prism 9.5.0 software and *p* values are indicated as asterisks in the graph (*:p <0.05, **: p < 0.005, ***: p <0.0005, ****: p<0.0001).

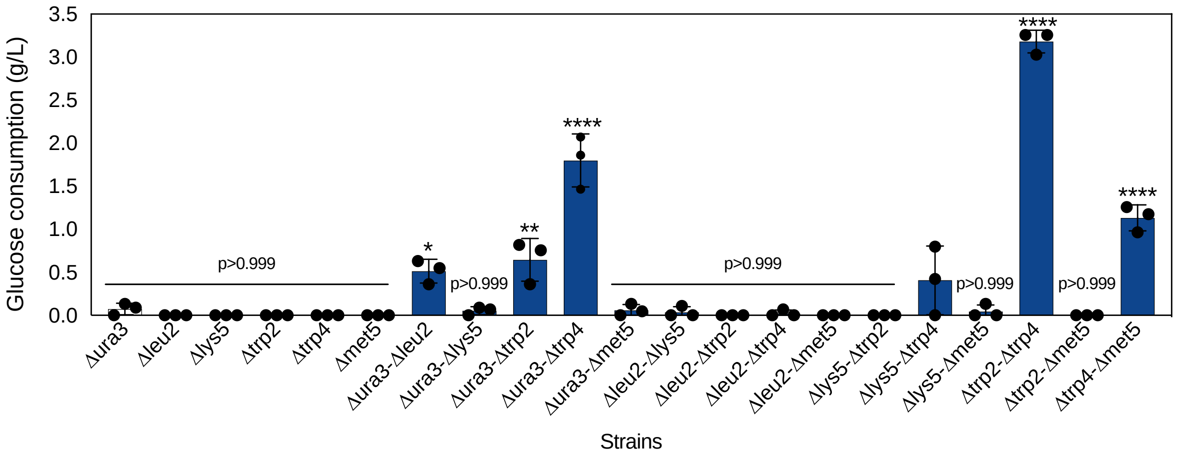

Supplementary Figure 4. Glucose consumption of selected syntrophic coculture of *Y. lipolytica* auxotroph strains at different inoculation ratio from 10:1 to 1:10. Values represent averages and error bars denote standard deviation (n=3).

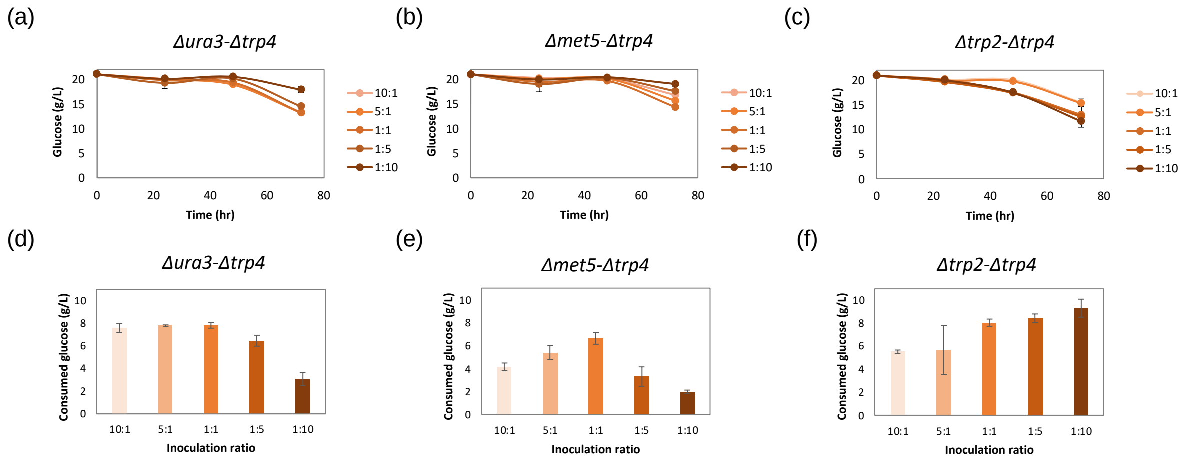

Supplementary Figure 5. Population of co-culture between YL*Δura3* and YL*Δtrp4* at different inoculation ratios. The populations were measured by Flow Cytometry and corresponding fluorescence microscope images were taken at 120 hours of cultivation. Values represent averages and error bars denote standard deviation (n=3).

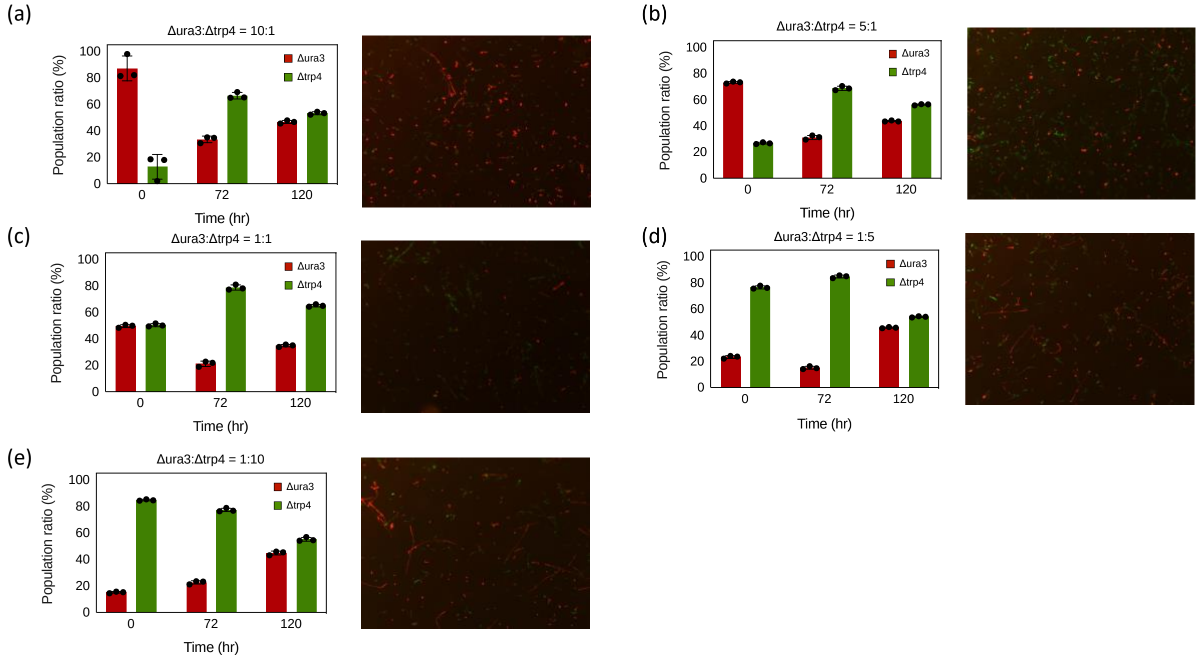

Supplementary Figure 6. Population of co-culture between YL*Δmet5* and YL*Δtrp4* at different inoculation ratios. The populations were measured by Flow Cytometry and corresponding fluorescence microscope images were taken at 120 hours of cultivation. Values represent averages and error bars denote standard deviation (n=3).

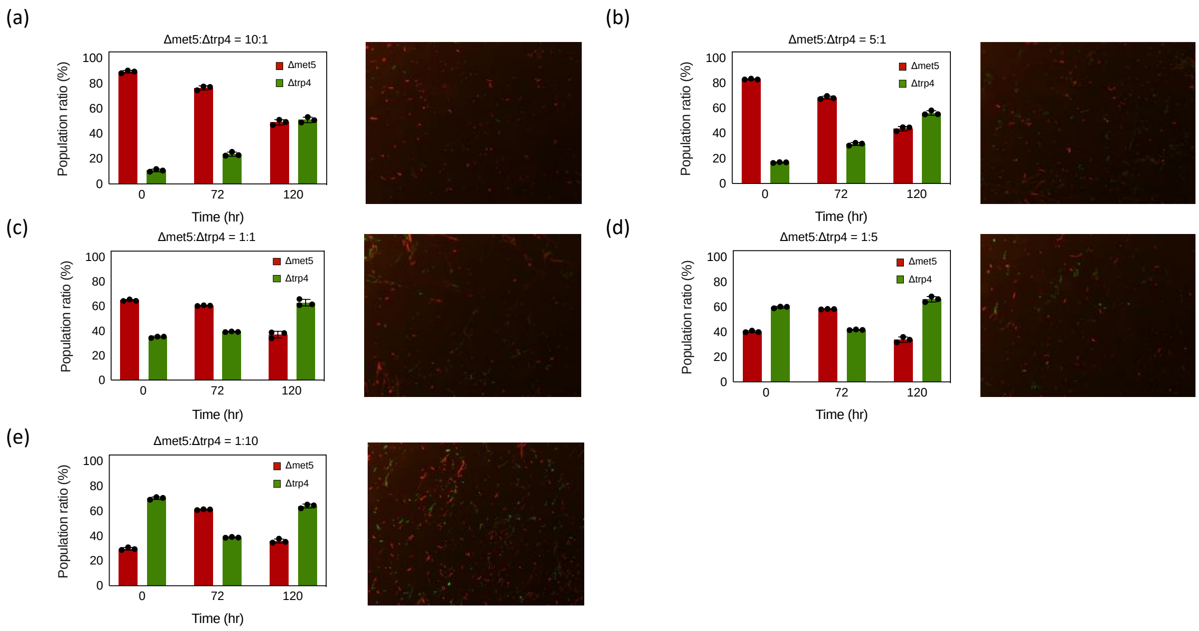

Supplementary Figure 7. Population of co-culture between YL*Δtrp2* and YL*Δtrp4* at different inoculation ratios. The populations were measured by Flow cytometry and corresponding fluorescence microscope images were taken at 120 hours of cultivation. Values represent averages and error bars denote standard deviation (n=3).

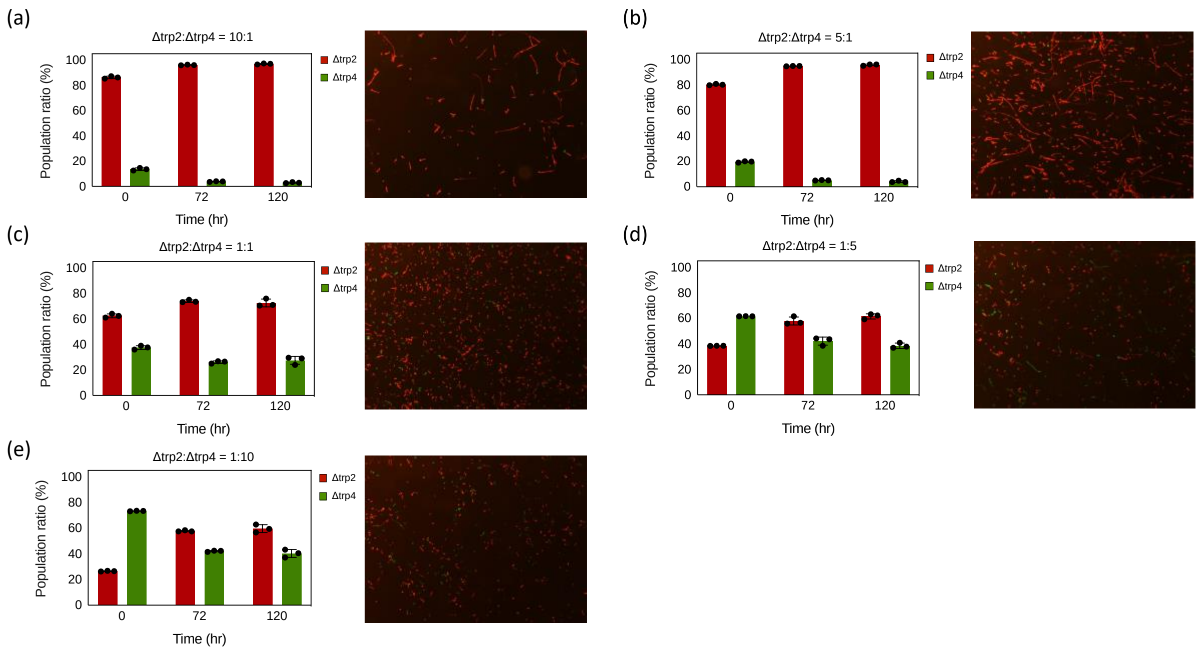

Supplementary Figure 8. Growth profile of interspecies syntrophic coculture between *Y. lipolytica* and *S. cerevisiae* (inoculation ratio = 1:1). (a) monoculture of *Y. lipolytica* auxotroph strains, (b) monoculture of *S. cerevisiae* auxotroph strain, (c) coculture pairs of *Δtrp2* and *Δtrp4*, (d) coculture pairs of *Δmet5* and *Δtrp4*, and (e) coculture pairs of *Δlys5* and *Δtrp4.* Values represent averages and error bars denote standard deviation (n=3).

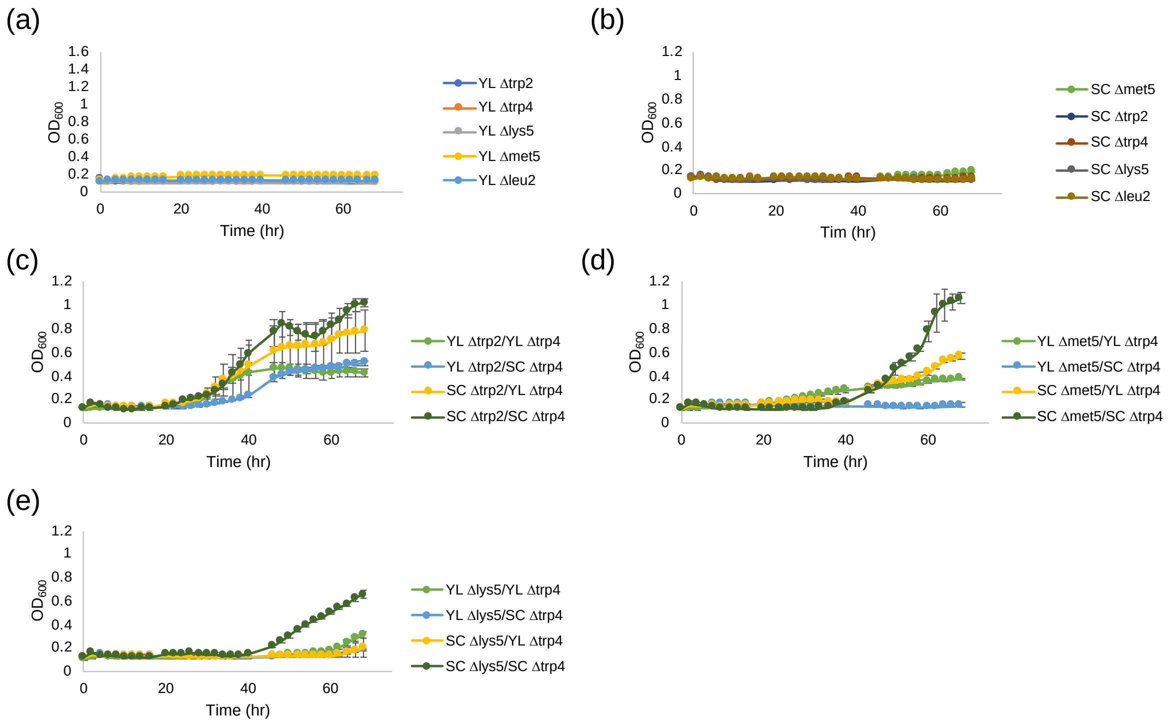

Supplementary Figure 9. Population of co-culture among YL*Δtrp2*, YL*Δtrp4*, SC*Δtrp2*, and SC*Δtrp4* at different inoculation ratios. The populations were measured by Flow Cytometry and corresponding fluorescence microscope images were taken at 120 hours of cultivation. Values represent averages and error bars denote standard deviation (n=3).

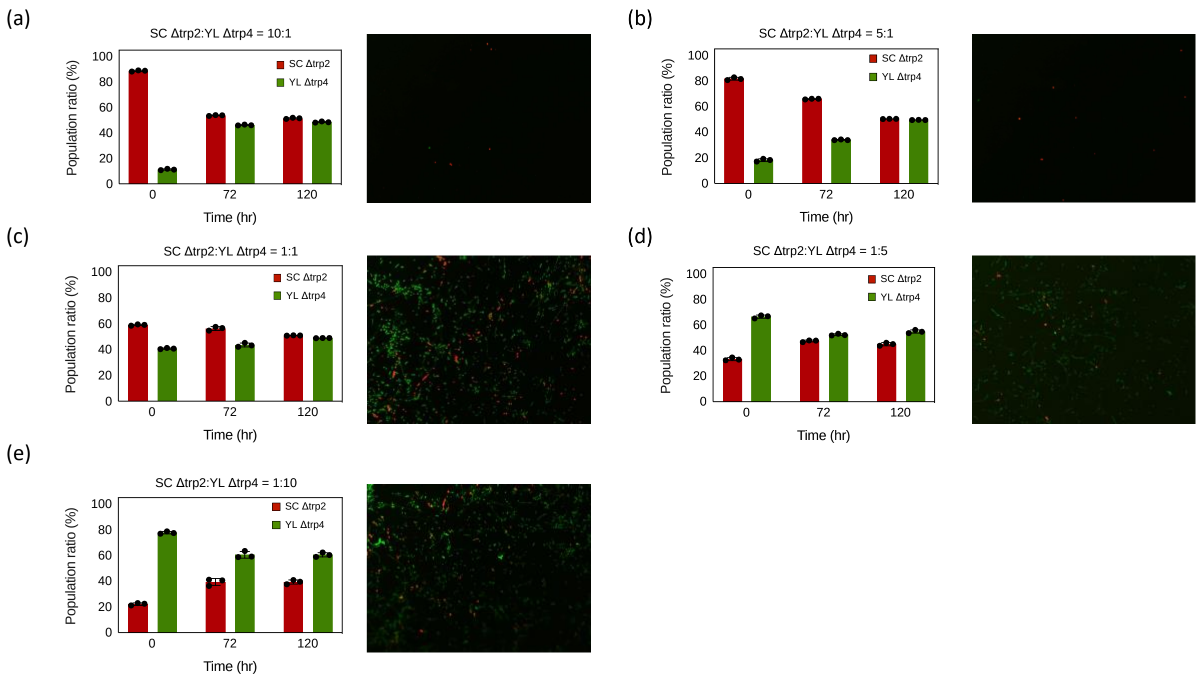

Supplementary Figure 10. Profiles of metabolites in co-culture among YL*Δtrp2*, YL*Δtrp4*, SC*Δtrp2*, and SC*Δtrp4* at different inoculation ratios in aerobic condition. Metabolites (glucose, ethanol, citrate, and glycerol) from the co-culture of (a-d) YL*Δtrp2* - YL*Δtrp4* and (e-h) SC*Δtrp2* - YL*Δtrp4*. Values represent averages and error bars denote standard deviation (n=3).

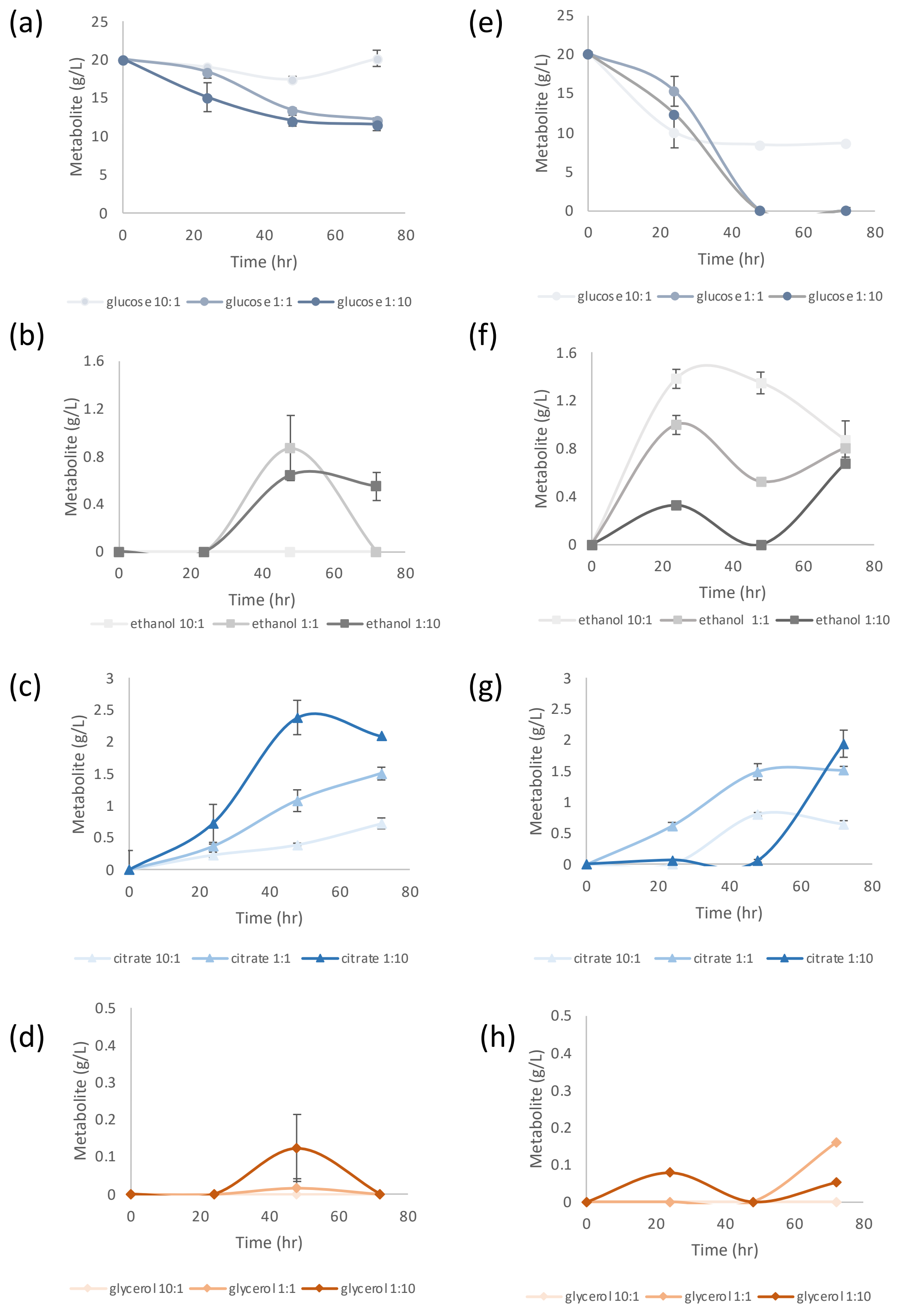

Supplementary Figure 11. Profiles of growth and metabolites in co-culture among YL*Δtrp2*, YL*Δtrp4*, SC*Δtrp2*, and SC*Δtrp4* at different inoculation ratios in different culture condition. (a) Aerobic condition (continuous shaking), and (b) semi-anaerobic condition (static and closed condition). Values represent averages and error bars denote standard deviation (n=3).

(a)

Glucose	20 g/L	100 g/L	20 g/L	100 g/L
Co-culture	YLΔtrp2-YLΔtrp4	YLΔtrp2-YLΔtrp4	SCΔtrp2-YLΔtrp4	SCΔtrp2-YLΔtrp4
Aeration	aerobic	aerobic	aerobic	aerobic

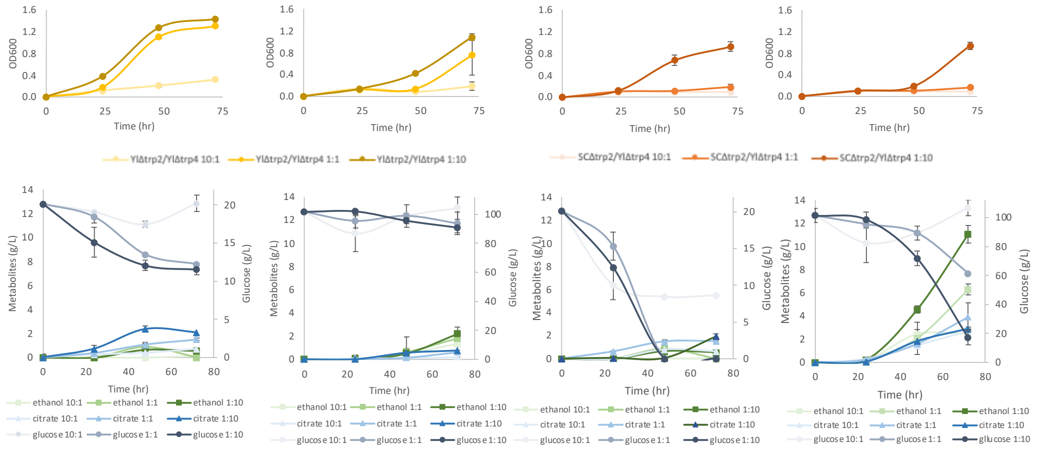

(b)

Glucose	20 g/L	100 g/L	20 g/L	100 g/L
Co-culture	YLΔtrp2-YLΔtrp4	YLΔtrp2-YLΔtrp4	SCΔtrp2-YLΔtrp4	SCΔtrp2-YLΔtrp4
Aeration	Semi-anaerobic	Semi-anaerobic	Semi-anaerobic	Semi-anaerobic

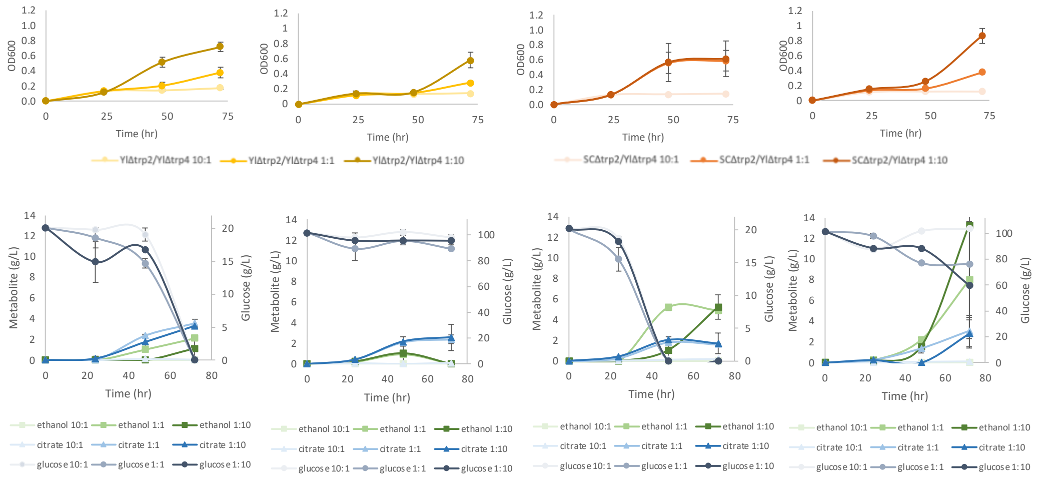

Supplementary Figure 12. Division of labor in syntrophic community of *Y. lipolytica* for bioproduction of 3-hydroxypropionic acid. (a) Growth of monoculture and cocultures of pairs with *Δtrp2* and *Δtrp4* at different inoculation ratios, (b) production of metabolites in the 3-HP synthetic pathway from monoculture and cocultures. The strains were incubated in flask at 30 °C with 250 rpm for 120 hours. Values represent averages and error bars denote standard deviation (n=2).

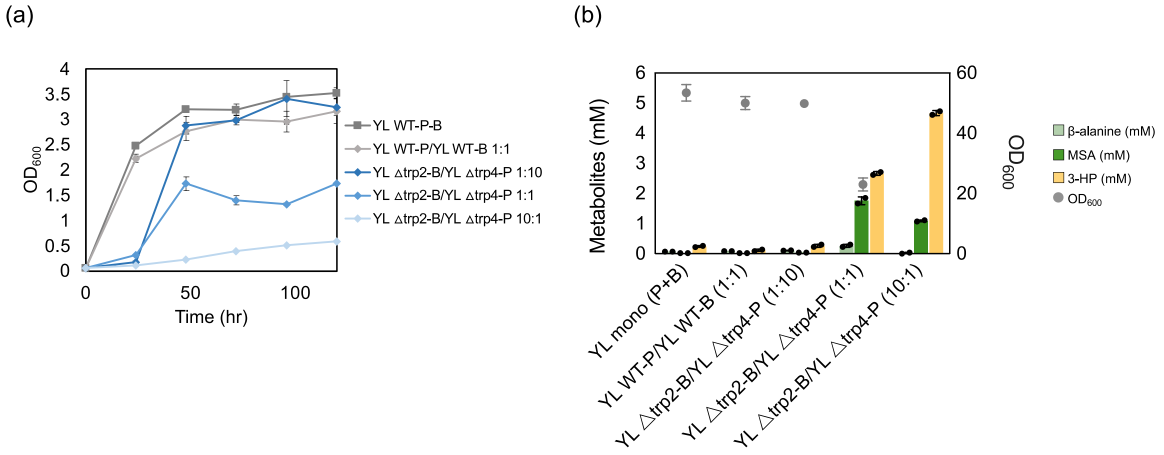

Supplementary Figure 13. Division of labor in interspecies syntrophic community of *Y. lipolytica* and *S. cerevisiae* for bioproduction of 3-hydroxypropionic acid. (a) Growth of interspecies cocultures of pairs with *Δtrp2* and *Δtrp4* at different inoculation ratios, (b) production of metabolites in the 3-HP synthetic pathway from monoculture and cocultures. The strains were incubated in flask at 30 °C with 250 rpm for 120 hours. Values represent averages and error bars denote standard deviation (n=2).

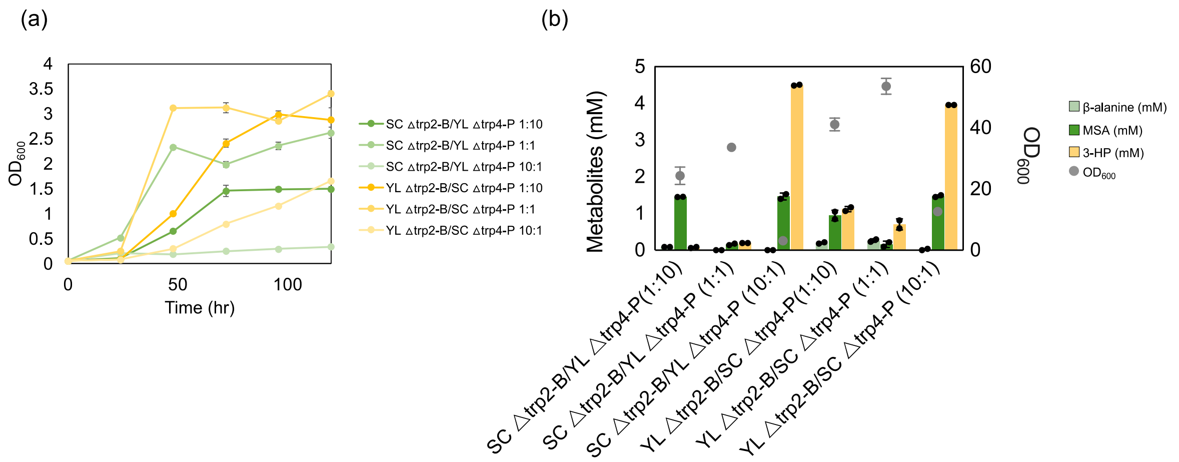

Supplementary Figure 14. Division of labor in syntrophic community of *S. cerevisiae* for bioproduction of 3-hydroxypropionic acid. (a) Growth of monoculture and cocultures of pairs with *Δtrp2* and *Δtrp4* at different inoculation ratios, (b) production of metabolites in the 3-HP synthetic pathway from monoculture and cocultures. The strains were incubated in flask at 30 °C with 250 rpm for 120 hours. Values represent averages and error bars denote standard deviation (n=2).

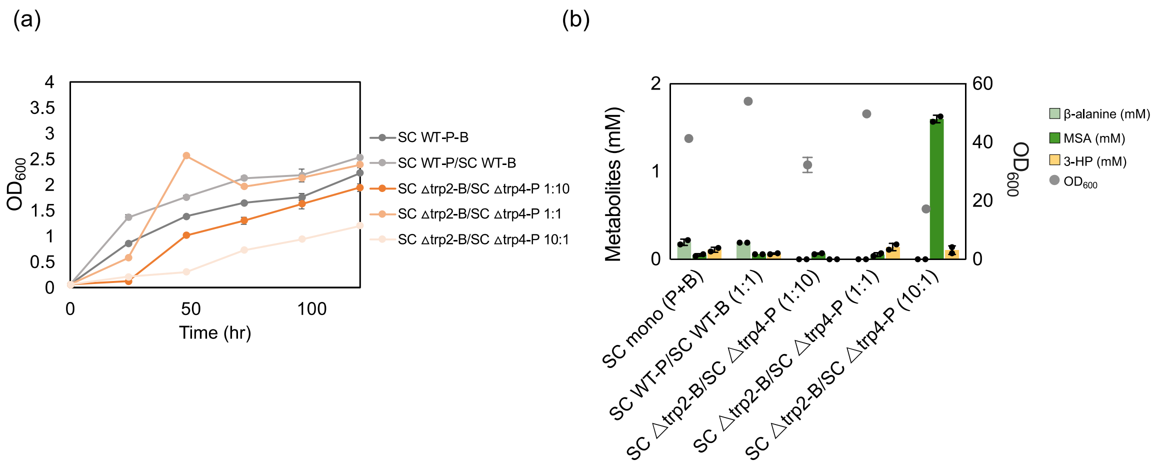

Supplementary Figure 15. Profiles of glucose consumption and byproduct formation in the co-cultures for 3-HP production. The strains were incubated in flask at 30 °C with 250 rpm for 120 hours. Values represent averages and error bars denote standard deviation (n=2).

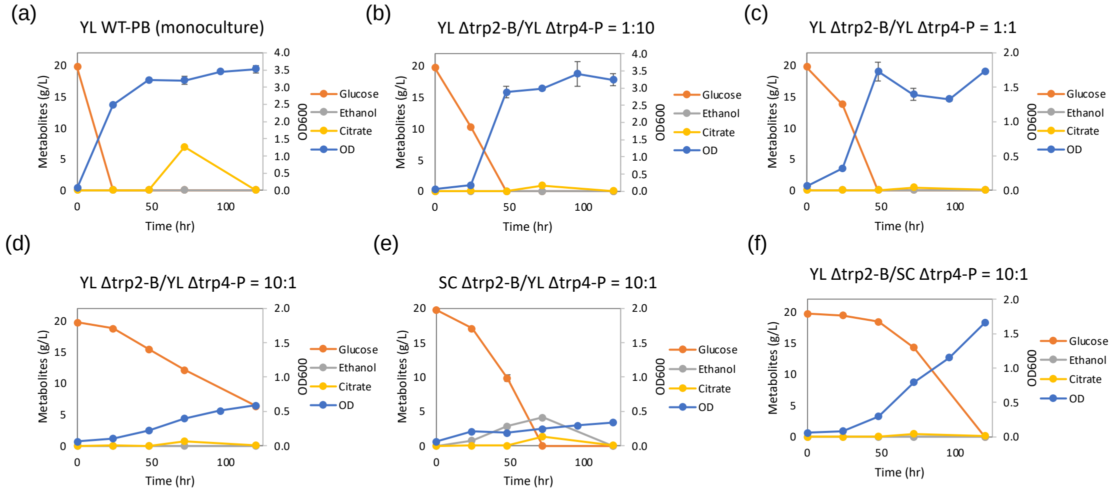

Supplementary Figure 16. (a) Metabolic pathway including synthetic 3-HP pathway (b) Production of metabolites (citrate, pyruvate, β-alanine, malonic semialdehyde, and 3-hydroxypropionic acid) from mono- and co-cutlures. The strains were incubated in flask at 30 °C with 250 rpm for 120 hours. Values represent averages and error bars denote standard deviation (n=2). One-way ANOVA, followed by Bonferroni's multiple comparisons test with 95% confidence intervals were performed using GraphPad Prism 9.5.0 software and *p* values are indicated as asterisks in the graph (*:*p* <0.05, **: *p* < 0.005, ***: *p* <0.0005, ****: *p*<0.0001).

(a)

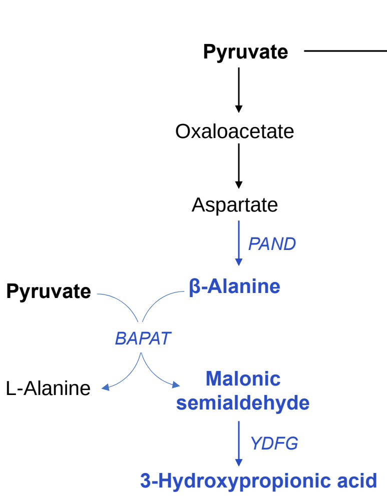

(b)

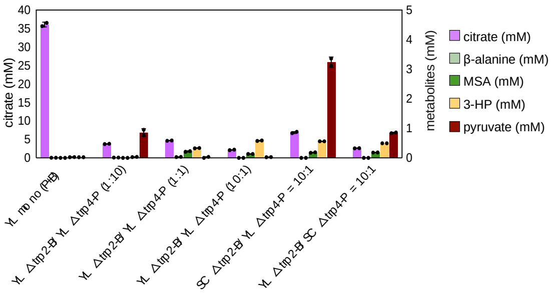

Supplementary Figure 17. Calibration curve of OD_{600} between microplate reader and spectrophotometer. The OD_{600} from spectrophotometer was calibrated to the value of microplate reader in figures in the main manuscript and Supplementary Figures.

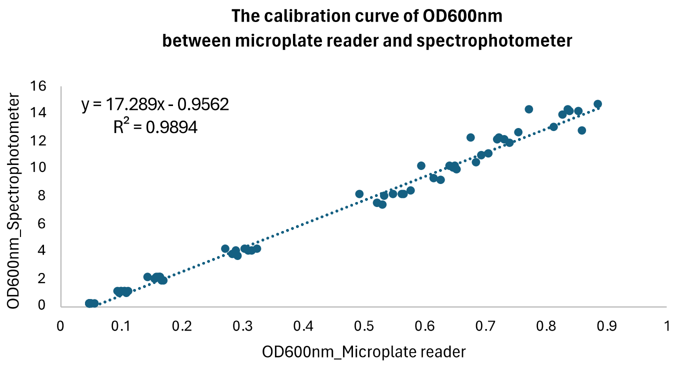

Supplementary Figure 18. Comparison of division of labor in the *S. cerevisiae* synthetic community between previous study and this study. Supplementary Data 8 of previous study (Aulakh et al. 2013) was used.

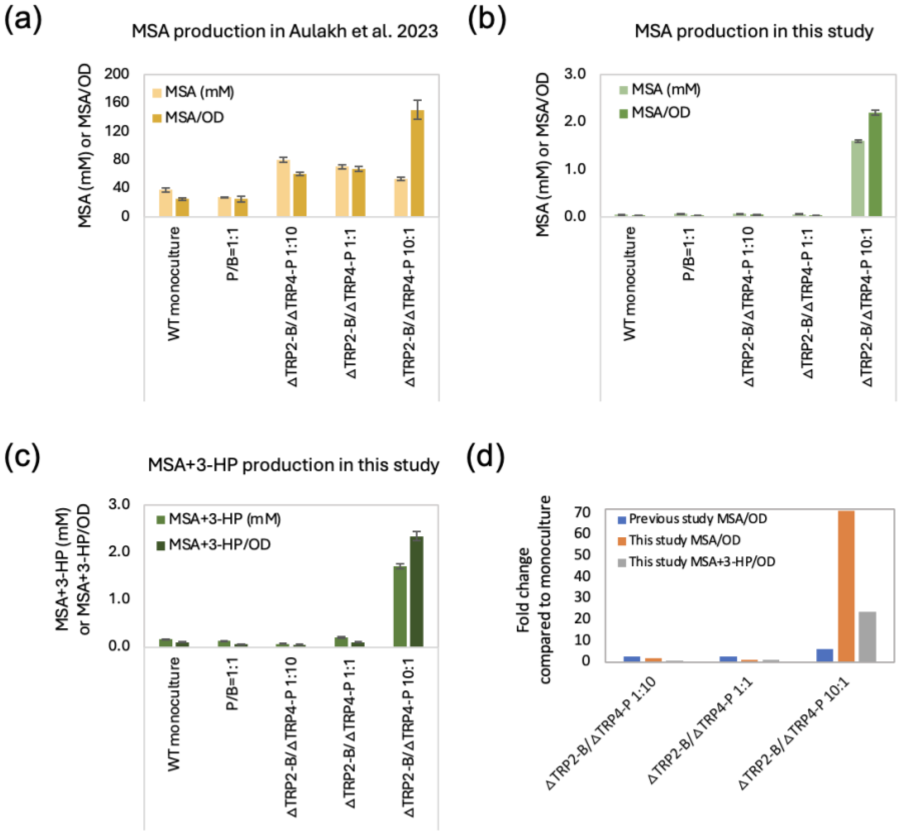

Supplement: Supplementary file 1 — Supplementary Information [file 41467_2024_53117_MOESM1_ESM.pdf]
