# Supplementary material for: Engineered cross-feeding creates inter- and intra-species synthetic yeast communities with enhanced bioproduction
Source: Nat Commun. 2024 Oct 16;15:8924. doi: 10.1038/s41467-024-53117-4 (PMC11484764; doi:10.1038/s41467-024-53117-4)
Supplement: Supplementary file 2 — Reporting Summary [file 41467_2024_53117_MOESM2_ESM.pdf]

nature portfolio

Corresponding author(s): Rodrigo Ledesma-Amaro
Last updated by author(s): Sep 11, 2024

# Reporting Summary

Nature Portfolio wishes to improve the reproducibility of the work that we publish. This form provides structure for consistency and transparency in reporting. For further information on Nature Portfolio policies, see our Editorial Policies and the Editorial Policy Checklist.

## Statistics

For all statistical analyses, confirm that the following items are present in the figure legend, table legend, main text, or Methods section.

| n/a | Confirmed | |
|---|---|---|
| ☐ | ☒ | The exact sample size (*n*) for each experimental group/condition, given as a discrete number and unit of measurement |
| ☐ | ☒ | A statement on whether measurements were taken from distinct samples or whether the same sample was measured repeatedly |
| ☐ | ☒ | The statistical test(s) used AND whether they are one- or two-sided<br>*Only common tests should be described solely by name; describe more complex techniques in the Methods section.* |
| ☒ | ☐ | A description of all covariates tested |
| ☒ | ☐ | A description of any assumptions or corrections, such as tests of normality and adjustment for multiple comparisons |
| ☐ | ☒ | A full description of the statistical parameters including central tendency (e.g. means) or other basic estimates (e.g. regression coefficient) AND variation (e.g. standard deviation) or associated estimates of uncertainty (e.g. confidence intervals) |
| ☐ | ☒ | For null hypothesis testing, the test statistic (e.g. *F*, *t*, *r*) with confidence intervals, effect sizes, degrees of freedom and *P* value noted<br>*Give P values as exact values whenever suitable.* |
| ☒ | ☐ | For Bayesian analysis, information on the choice of priors and Markov chain Monte Carlo settings |
| ☒ | ☐ | For hierarchical and complex designs, identification of the appropriate level for tests and full reporting of outcomes |
| ☒ | ☐ | Estimates of effect sizes (e.g. Cohen's *d*, Pearson's *r*), indicating how they were calculated |

*Our web collection on statistics for biologists contains articles on many of the points above.*

## Software and code

Policy information about availability of computer code

| | |
|---|---|
| Data collection | The raw data from LC-MS were processed using Agilent MassHunter Qualitative Analysis software. |
| Data analysis | 1. Microsoft Office 365 Excel and GraphPad Prism 9.5.0 software were used for data averaging, calculation of standard divations.<br>2. GraphPad Prism 9.5.0 software was used for the statistics.<br>3. Benchling was used for designing all nucleotide sequences.<br>4. Chromeleon software was used for analyzing HPLC raw data.<br>5. MassHunter Qualitative Analysis software (Agilent) was used for analyzing LC-MS raw data. |

For manuscripts utilizing custom algorithms or software that are central to the research but not yet described in published literature, software must be made available to editors and reviewers. We strongly encourage code deposition in a community repository (e.g. GitHub). See the Nature Portfolio guidelines for submitting code & software for further information.

## Data

Policy information about availability of data

All manuscripts must include a data availability statement. This statement should provide the following information, where applicable:

- Accession codes, unique identifiers, or web links for publicly available datasets
- A description of any restrictions on data availability
- For clinical datasets or third party data, please ensure that the statement adheres to our policy

The authors declare that the data supporting the findings of this study are available within the paper and its Supplementary Information.

## Research involving human participants, their data, or biological material

Policy information about studies with human participants or human data. See also policy information about sex, gender (identity/presentation), and sexual orientation and race, ethnicity and racism.

| | |
|---|---|
| Reporting on sex and gender | Not relevant for this study. |
| Reporting on race, ethnicity, or other socially relevant groupings | Not relevant for this study. |
| Population characteristics | Not relevant for this study. |
| Recruitment | Not relevant for this study. |
| Ethics oversight | Not relevant for this study. |

Note that full information on the approval of the study protocol must also be provided in the manuscript.

# Field-specific reporting

Please select the one below that is the best fit for your research. If you are not sure, read the appropriate sections before making your selection.

- [x] Life sciences
- [ ] Behavioural & social sciences
- [ ] Ecological, evolutionary & environmental sciences

For a reference copy of the document with all sections, see nature.com/documents/nr-reporting-summary-flat.pdf

# Life sciences study design

All studies must disclose on these points even when the disclosure is negative.

| | |
|---|---|
| Sample size | No samplesize calculation was determined. N=2 or N=3 biological replicates were tested to provide sufficient reproducibility. The design was based on prior assay experience and similar experiments reported in the literature. |
| Data exclusions | No data were excluded for analysis. |
| Replication | Data are presented as mean values and standard deviations of two or three independent biological replicates. All attemps shown in this study were successful. |
| Randomization | This is not relevant to our study. As we carried out metabolic engineering on a specific strain and used the engineered strains for fermentations, randomization is not applicable. |
| Blinding | Blinding does not apply to our experiments. Experiments are rationally designed to achieve our goal. |

# Reporting for specific materials, systems and methods

We require information from authors about some types of materials, experimental systems and methods used in many studies. Here, indicate whether each material, system or method listed is relevant to your study. If you are not sure if a list item applies to your research, read the appropriate section before selecting a response.

## Materials & experimental systems

| n/a | Involved in the study |
|---|---|
| ☒ | ☐ Antibodies |
| ☒ | ☐ Eukaryotic cell lines |
| ☒ | ☐ Palaeontology and archaeology |
| ☒ | ☐ Animals and other organisms |
| ☒ | ☐ Clinical data |
| ☒ | ☐ Dual use research of concern |
| ☒ | ☐ Plants |

## Methods

| n/a | Involved in the study |
|---|---|
| ☒ | ☐ ChIP-seq |
| ☐ | ☒ Flow cytometry |
| ☒ | ☐ MRI-based neuroimaging |

# Plants

| | |
|---|---|
| Seed stocks | Not relevant for this study. |
| Novel plant genotypes | Not relevant for this study. |
| Authentication | Not relevant for this study. |

# Flow Cytometry

## Plots

Confirm that:

☒ The axis labels state the marker and fluorochrome used (e.g. CD4-FITC).

☒ The axis scales are clearly visible. Include numbers along axes only for bottom left plot of group (a 'group' is an analysis of identical markers).

☒ All plots are contour plots with outliers or pseudocolor plots.

☒ A numerical value for number of cells or percentage (with statistics) is provided.

## Methodology

| | |
|---|---|
| Sample preparation | Flow cytometry was used for population analysis. |
| Instrument | Attune Nxt flow cytometer (Thermo Scientific) was used. |
| Software | Attune Cytometric software was used for data collection. |
| Cell population abundance | Fluorescence data was collected from at least 10,000 cells for each experiment with three biological replicates. |
| Gating strategy | Yeast cells were gated fro singlets using FSC-H vs FSC-A and to remove background noise. No other Gatings was performed on global yeast population. > 10,000 events were collected and anlyzed within the singlets gate from each measurement. |

☒ Tick this box to confirm that a figure exemplifying the gating strategy is provided in the Supplementary Information.
